# Fire‐Pollutant‐Atmosphere Components and Its Impact on Mortality in Portugal During Wildfire Seasons

**DOI:** 10.1029/2023GH000802

**Published:** 2023-10-06

**Authors:** Ediclê de Souza Fernandes Duarte, Vanda Salgueiro, Maria João Costa, Paulo Sérgio Lucio, Miguel Potes, Daniele Bortoli, Rui Salgado

**Affiliations:** ^1^ Instituto de Ciências da Terra—ICT (Pólo de Évora) Instituto de Investigação e Formação Avançada (IIFA) Universidade de Évora Évora Portugal; ^2^ Earth Remote Sensing Laboratory (EaRSLab) Instituto de Investigação e Formação Avançada (IIFA) Universidade de Évora Évora Portugal; ^3^ Departamento de Física Escola de Ciências e Tecnologia (ECT) Universidade de Évora Évora Portugal; ^4^ Departamento de Ciências Atmosféricas e Climáticas Universidade Federal do Rio Grande do Norte Natal Brazil

**Keywords:** atmospheric pollutants, environmental health, cardio‐respiratory mortality, environmental risks, multivariate statistical techniques

## Abstract

This study analyzed fire‐pollutant‐meteorological variables and their impact on cardio‐respiratory mortality in Portugal during wildfire season. Data of burned area, particulate matter with a diameter of 10 or 2.5 μm (μm) or less (PM_10_, PM_2.5_), carbon monoxide (CO), nitrogen dioxide (NO_2_), ozone (O_3_), temperature, relative humidity, wind speed, aerosol optical depth and mortality rates of Circulatory System Disease (CSD), Respiratory System Disease (RSD), Pneumonia (PNEU), Chronic Obstructive Pulmonary Disease, and Asthma (ASMA), were used. Only the months of 2011–2020 wildfire season (June–July–August–September‐October) with a burned area greater than 1,000 ha were considered. Principal component analysis was used on fire‐pollutant‐meteorological variables to create two indices called Pollutant‐Burning Interaction (PBI) and Atmospheric‐Pollutant Interaction (API). PBI was strongly correlated with the air pollutants and burned area while API was strongly correlated with temperature and relative humidity, and O_3_. Cluster analysis applied to PBI‐API divided the data into two Clusters. Cluster 1 included colder and wetter months and higher NO_2_ concentration. Cluster 2 included warmer and dried months, and higher PM_10_, PM_2.5_, CO, and O_3_ concentrations. The clusters were subjected to Principal Component Linear Regression to better understand the relationship between mortality and PBI‐API indices. Cluster 1 showed statistically significant (*p*‐value < 0.05) correlation (*r*) between RSDxPBI (*r*_RSD_ = 0.58) and PNEUxPBI (*r*_PNEU_ = 0.67). Cluster 2 showed statistically significant correlations between RSDxPBI (*r*_RSD_ = 0.48), PNEUxPBI (*r*_PNEU_ = 0.47), COPDxPBI (*r*_COPD_ = 0.45), CSDxAPI (*r*_CSD_ = 0.70), RSDxAPI (*r*_CSD_ = 0.71), PNEUxAPI (*r*_PNEU_ = 0.49), and COPDxAPI (*r*_PNEU_ = 0.62). Cluster 2 analysis indicates that the warmest, driest, and most polluted months of the wildfire season were associated with cardio‐respiratory mortality.

## Key Points

- Combining fire‐pollutant‐meteorological data can be an effective way to understand how these combined hazards can affect health outcomes
- The hottest, driest, and most polluted months of the fire season were associated with higher cardio‐respiratory mortality
- Colder and wetter months within the wildfire season were not associated with increased or decreased cardio‐respiratory mortality

## 1 Introduction

Exposure to poor air quality increases morbidity and mortality contributing significantly to the global burden of disease (Cohen et al., 2017). Air pollution—both household and ambient—remains responsible for 6.7 million deaths in 2019 (Fuller et al., 2022). In Europe, air pollution is the largest environmental risk and has a significant impact on the health of the European population (EEA, 2020). A significant proportion of premature deaths in Europe could be avoided annually if air pollution concentrations were reduced, particularly below World Health Organization (WHO) guidelines (Khomenko et al., 2021).

In Europe, important sources of air pollution are emissions from transportation, domestic heating, energy production, and industrial combustion (Malico et al., 2017), although emissions from wildfires during the fire season can significantly degrade air quality, as well as impact climate in different ways (Cattani et al., 2006; Santos et al., 2008). Wildfires emit large amounts of air pollutants that can be transported far from the source of origin affecting the air quality and human health (Augusto et al., 2020; Bowman et al., 2017; E. S. F. Duarte et al., 2021; Hua et al., 2014; Janssen et al., 2012; Machado‐Silva et al., 2020; Requia et al., 2021; Tarín‐Carrasco et al., 2021; Youssouf et al., 2014). Nevertheless, the combination of extreme drought and heat waves has been identified as a crucial factor for the occurrence of wildfires in Mediterranean forests and scrublands, leading to significant socioeconomic impacts (Ruffault et al., 2020) such as burn timber, make recreation and tourism unappealing, and affect agricultural production. Heat stress (high‐temperature driven hazards) and wildfires are often considered highly correlated hazards, as extreme temperatures play a key role in both events (Sutanto et al., 2020; Vitolo et al., 2019).

On the other hand, emissions from wildfires can exacerbate the effects of heat stress on the human body, particularly in the cardiovascular and respiratory systems (Finlay et al., 2012). Primary emissions from wildfires that degrade air quality include particulate matter (PM_2.5_ and PM_10_), black carbon (BC), and gaseous substances such as carbon monoxide (CO), methane (CH_4_), nitrous oxide (N_2_O), and other combustion pollutants (Urbanski et al., 2008). Air pollution from biomass burning also contributes to the formation of secondary pollutants such as polycyclic aromatic hydrocarbons and volatile organic compounds (VOCs), as well as ozone (O_3_) formed by the photoreaction of nitrous oxides (NO_*x*_) in the atmosphere (Jaffe & Wigder, 2012).

Climate has a strong influence on global wildfire activity, with the frequency and intensity of wildfires increasing in many regions due to climate change (Couto et al., 2022; Jolly et al., 2015; Moritz et al., 2014). Wildfires occur at the intersection of dry weather, available biomass fuel and ignition sources (Moritz et al., 2005). According to Abatzoglou et al. (2013), weather conditions are the most important factors in regional fire extent. Meteorological variables such as temperature, relative humidity, precipitation, and wind speed independently influence the rate and intensity of wildfire spread. On the other hand, the coincidence of multiple weather extremes, such as the simultaneous occurrence of hot, dry, and windy conditions, results in more severe fires (Couto et al., 2020; Flannigan & Harrington, 1988). Several studies suggest that the coincidence of drought and high temperatures promotes larger fires in southern Europe (Chuvieco, 2009; Pausas, 2004; Pausas et al., 2008; Pereira et al., 2005, 2011; Trigo et al., 2016; Turco et al., 2013, 2017, 2018, 2019; Viegas & Viegas, 1994).

Regarding the health effects of the exposure to wildfire smoke, epidemiologic studies showed an association between the exposure to wildfire smoke and the respiratory morbidity, with increasing evidence of an association with all‐cause mortality (Reid et al., 2016). Pollutants from wildfires are a risk factor for adverse cardiovascular outcomes, particularly in vulnerable populations such as the elderly, pregnant women, and those of low socioeconomic status (Chen et al., 2021). Young and healthy individuals may also develop biological responses, including systemic inflammation and vascular activation (Chen et al., 2021). In Europe, several studies have been conducted on the health effects of population exposure to wildfire smoke (Augusto et al., 2020; Barbosa et al., 2022; Brito et al., 2021; Chas‐Amil et al., 2020; European Commission, 2020; Faustini et al., 2015; Hänninen et al., 2009; Linares et al., 2018; Oliveira et al., 2020; Tarín‐Carrasco et al., 2021; Youssouf et al., 2014). In all these works, different methods were used to show the importance of wildfires in Europe during the fire season as a public health problem.

Because Portugal is a highly fire‐prone region due to existing vegetation and favorable weather conditions, further epidemiological studies on smoke exposure are essential. On the other hand, air pollution released by wildfires can be transported over long distances (Baars et al., 2019; Osborne et al., 2019; Salgueiro et al., 2021; Sicard et al., 2019), putting multiple populations at risk. In addition, wildfires in Portugal have a significant impact on air quality throughout Europe (Augusto et al., 2020; Brito et al., 2021; Tarín‐Carrasco et al., 2021; Turco et al., 2019). Another important factor is that Portugal has an increasing elderly population—a group more prone to developing health problems and more vulnerable to weather extremes and the effects of climate change—and a decreasing younger population, according to INE (2022). Previous studies have found wildfire‐related air pollution to be significantly associated with increased mortality risk (Augusto et al., 2020; Tarín‐Carrasco et al., 2021).

This work aims at evaluating the correlation between fire‐pollutant‐meteorology components and the mortality in Portugal during the wildfire seasons of 2011–2020. Fire‐pollutant‐meteorological components refers to a hazardous condition or situation that arises from the combination of fire, pollutants, and meteorological factors. Evaluating the correlation between these components and cardio‐respiratory mortality can provide valuable insights into the health impacts of wildfires and associated pollution. To this end, the effects of the PM_10_, PM_2.5_, CO, NO_2_, O_3_, temperature, relative humidity, wind speed, burned area, and aerosol optical depth (AOD) on mortality rates due to Circulatory System Disease (CSD), Respiratory System Disease (RSD), Pneumonia (PNEU), Chronic Obstructive Pulmonary Disease (COPD), and Asthma (ASMA) are investigated using multivariate statistical methods. Because small wildfires do not have a significant effect on mortality rates (Analitis et al., 2012), only the fire season months (June, July, August, September, and October) with a burned area greater than 1,000 ha were considered in this study. With the objective of increasing the knowledge of the effects of fire, pollutants, and meteorological variables on health outcome, this study examines how the combination of these factors affects the mortality of the country's population during fire season. By investigating the effects of various environmental variables (fire‐pollutant‐meteorological components), the study aims to identify the relationships between these factors and mortality rates during the wildfire seasons in Portugal.

## 2 Materials and Methods

### 2.1 Study Area

**Figure 1:**
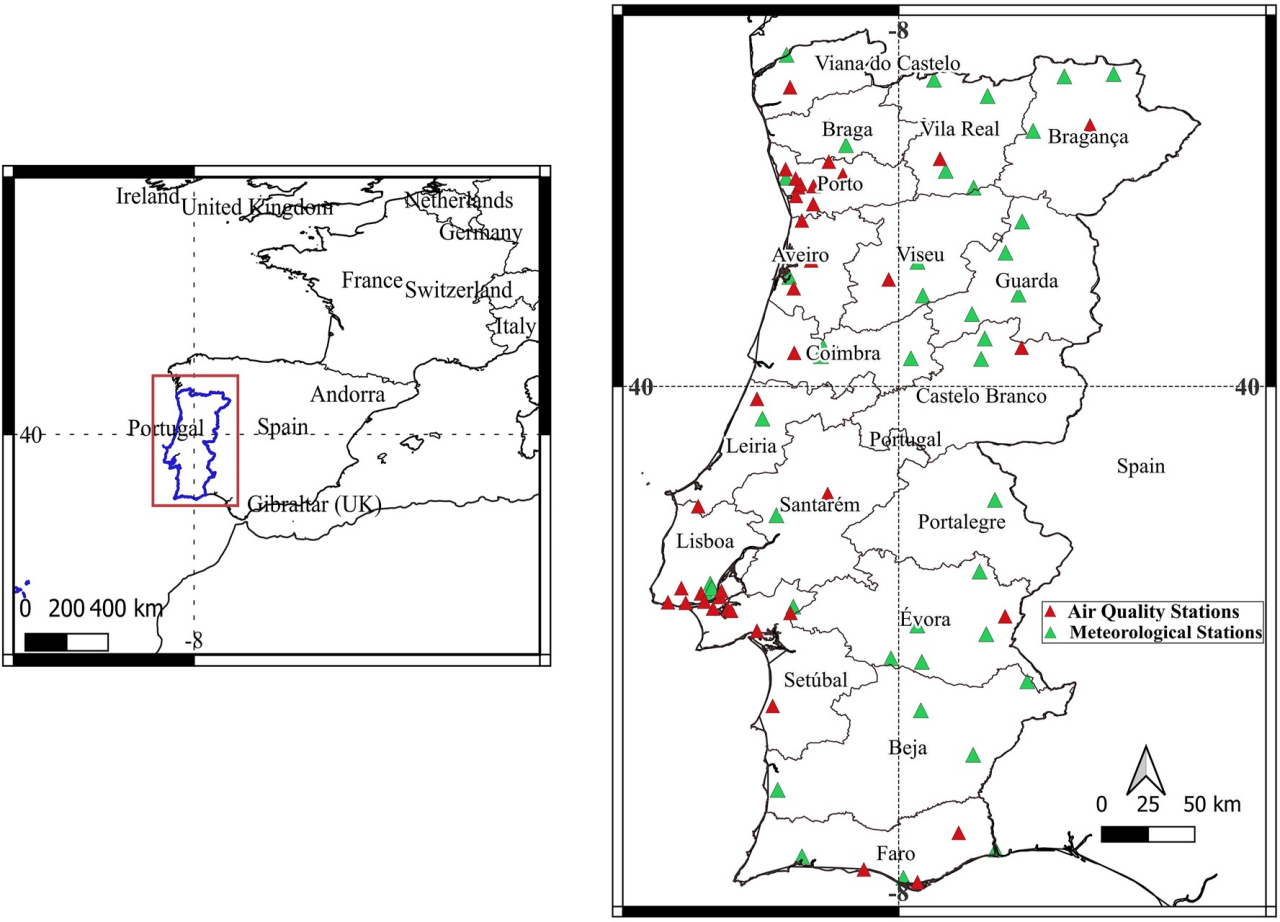
Location of Portugal in Western Iberia and the location of the 18 Portuguese continental administrative regions (districts). The map displays the background air quality stations (red triangles) and meteorological stations (green triangles) used in this work.

Portugal is located in southwestern Europe, on the Iberian Peninsula, facing the Atlantic Ocean on its west and south coasts (Figure 1), in the transition zone between subtropical and mid‐latitude climates. The study site was strategically chosen because the population and ecosystems frequently suffer from intense natural hazards such as droughts, heat waves, and wildfires, which tend to become more intense and frequent under climate change (Turco et al., 2019). Continental Portugal has a temperate Mediterranean hot summer climate (Csa) in the south and a Mediterranean mild summer climate (Csb) in much of the north, with a small area with a mid‐latitude steppe (BSk) climate. Figure 1 shows the background air quality (red triangles) stations as well as meteorological (green triangles) stations used in this work. All the data used on this work are in Supporting Information S1.

### 2.2 Burned Area, Air Pollution and Meteorological Data

The burned area (Burned_Area; ha) data were obtained from the Portuguese Institute of Nature and Forest Conservation (https://www.icnf.pt/). These data correspond to monthly burned area data taken from 2011 to 2020 in Continental Portugal. The burned area is obtained based on ground and satellite measurements according to the detailed information on the date and time of ignition and extinction of fire events (Pereira et al., 2011) and assessment of changes in fire regime due to different climate and fire management activities (Parente et al., 2016, 2019).

Air pollution data were obtained from the online air quality database (QualAR) of the Portuguese Environmental Agency (APA; https://qualar.apambiente.pt). The QualAR air pollution database also contains information on the type of station based on their locations (urban, suburban, and rural) and the type of emission impact (background, transport, and industrial), according to the Commission Decision 2001/752/EC of 17 October 2001 (APA, 2008). The background stations are in geographic areas far from the influence of transportation routes, industrial areas, or other anthropogenic sources, making them a good tool for assessing wildfire impacts. The air quality network APA monitors pollutant concentrations in accordance with the requirements of European legislation (European Directive 2008/50/EC of 21 May 2008).

Data used here refer to PM_10_, PM_2.5_, CO, O_3_, and NO_2_ hourly concentrations measured at 41 background stations distributed over Portugal (see red triangles in Figure 1) from 2011 to 2020. From the hourly data, the daily and monthly mean concentrations were calculated. The national monthly mean concentrations of PM_10_, PM_2.5_, CO, O_3_, and NO_2_ were used as five variables named PM10_Obs, PM25_Obs, CO_Obs, O3_Obs, and NO2_Obs for multivariate statistical analysis. To note that there are some gaps in the QualAR network registered data for PM_10_, PM_2.5_, CO, O_3_, and NO_2_ since ambient air monitoring procedures were varied over the years (2011–2020). Months with more than 75% of valid daily data were considered valid and included in the analyses.

The daily mean concentrations of PM_10_, PM_2.5_, and NO_2_ were used to calculate the number of times that PM_10_, PM_2.5_, and NO_2_ exceeded the daily WHO (2021) global air quality guidelines 2021 (15 μg/m^3^ 24‐hr average for PM_2.5_, 45 μg/m^3^ 24‐hr average for PM_10_, and 25 μg/m^3^ 24‐hr average for NO_2_). Daily exceedances of the WHO guidelines for PM_10_, PM_2.5_, and NO_2_ were counted monthly and included as three variables named WHO_PM10, WHO_PM25, and WHO_NO2 for multivariate statistical analysis.

Meteorological data of air temperature at 2 m above the surface (TEMP_Obs) and relative humidity (RH_Obs), and 10 m wind speed (WS_Obs) from 43 meteorological stations in mainland Portugal (green triangles in Figure 1), were provided by the Portuguese Institute of the Sea and Atmosphere (IPMA; www.ipma.pt/) for the period between 2011 and 2020. From the hourly meteorological data, the daily and monthly mean were calculated. Months with more than 75% of valid daily data were considered valid and included in the analyses.

Another important source of data for this work was the European Center for Medium‐Range Weather Forecasts (ECMWF). The ECMWF operates services related to meteorology and atmospheric composition and the data are available through the Copernicus Atmosphere Monitoring Service (CAMS; https://ads.atmosphere.copernicus.eu) on behalf of the European Union, including those provided by the CAMS global reanalysis (EAC4) monthly averaged fields. A validation of the CAMS global reanalysis can be found in Inness et al. (2019). The CAMS global reanalysis combines models with in situ and remote sensing observations through data assimilation techniques. In this work, the CAMS global reanalysis monthly averaged fields of the Total AOD at 550 nm (AOD_CAMs), BC AOD at 550 nm (BC_AOD_CAMs) and Dust AOD at 550 nm (Dust_AOD_CAMs) were used. AOD is a widely used parameter derived from satellite‐based observations and defined as the integration of aerosol extinction into the total atmospheric column (Jiang et al., 2021). The monthly mean data were obtained with a spatial resolution of 0.75° (∼80 km) over Portugal for the fire seasons (June–July–August–September–October) of 2011–2020 and it was calculated the spatial mean of the grid points that were within the domain 44°N, −10°W, 35°S, −5°E, restricted area mainly over Portugal.

For this study only the months of June–July–August–September–October of 2011–2020 with a burned area greater than 1,000 ha were considered. The data are available in Table S1 in Supporting Information S1.

### 2.3 Health and Population Data

Monthly national mortality data for Portugal were provided by the National Institute of Statistics (INE) (INE; https://www.ine.pt/). These data refer to mortality from a specific cause in 2011–2020, based on the use of administrative data for statistical purposes from the Integrated System for Civil Registration and Identification (SIRIC) and the Information System for Death Certificates (SICO). Standardized mortality rates (per 100,000 inhabitants—all ages) were selected according to the International Classification of Diseases, version 11 (ICD‐11; https://icd.who.int/browse11/l-m/en): CSD (ICD‐11: 1B40–BE2Z); RSD (ICD‐11: CA00–CB40); PNEU (ICD‐11: CA40–CA40.Z); COPD (ICD‐11: CA20.Z–CA22.Z); and ASMA (ICD‐11: CA23–CA23.3). Because most data exhibit seasonal variation, monthly data were used to examine within‐year variability in environmental health data, focusing on the fire season in Portugal (June to October) for the period between 2011 and 2020. Since the monthly national mortality data from INE were not available by region or Nomenclature of Territorial Units for Statistics (NUTS), the used data corresponds to the entire mainland Portugal.

### 2.4 Statistical Analyses

The impact of fires‐pollutant‐meteorological components on the mortality rate was examined using intra‐annual analyzes of the fire seasons over the 10‐year period of 2011–2020. The standardized anomalies (*Z*) method was used to ensure that the different variables were weighted equally in the statistical analysis. Accordingly, the monthly values of each variable (*X*) are used to calculate their respective long term sample mean ($\underline{X}$) and standard deviation (*s*), and standardized anomalies (*Z*) for each month are then plotted as in Equation 1:

(1) $$Z=\frac{\left(X-\underline{X}\right)}{s}$$

Bartlett's sphericity and the Kaiser‐Meyer‐Olkin (KMO) tests were applied to determine the suitability of the fire‐pollutant‐meteorological data for Principal Component Analysis (PCA). Bartlett's sphericity tests the hypothesis that the sample correlation matrix comes from a multivariate normal population in which the variables of interest are independent. A rejection of the hypothesis is taken as an indication that the data are suitable for analysis. The KMO statistic is a Measure of Sampling Adequacy (Cerny & Kaiser, 1977; Dziuban & Shirkey, 1974; Kaiser, 1970).

A significance test was performed to derive a *p*‐value for the correlation coefficient between the variables by applying the function *corr.test*() (package *psych*; *R* software). The null hypothesis states that the correlation coefficient from which the sample was drawn is zero. The alternative hypothesis states that the correlation coefficient from which the sample was drawn is non‐zero. If the probability is less than the usual 5% (*p*‐value < 0.05), the correlation coefficient is called statistically significant. The correlation matrix presents the correlation between the variables. In the other hand, correlations between independent variables close to −1 or +1 might indicate the existence of collinearity between them. For these cases, It was used *ols_vif_tol()* function from the *olsrr* package from *R software* to calculate the Tolerance (TI) and the Variance Inflation Factor values (VIF). VIF and the closely related TI were used to estimates de degree of interrelationship of an independent variable with other explanatory variables before the application of the PCA. As a general guideline, a VIF ≥ 10 and a TI of <0.1 might indicate harmful collinearity. Collinearity between variables may affects the interpretability of the model, but not the ability of the model to predict or the accuracy of the model. The TI measures the percent of the variance in the independent variable that cannot be accounted for by the other independent variables.

The strength of the relationships between fire and atmospheric variables during the fire season in Portugal was assessed by a multivariate approach called Principal Component Analysis (PCA) based on the correlation matrix. Pearson correlation (*r*) measures a linear dependence between two variables. It is a parametric correlation test because it depends on the distribution of the data. Correlation test was used to evaluate the association between the variables. For the Pearson correlation, the variables should be normally distributed. Other assumptions include linearity and homoscedasticity. Linearity assumes a straight‐line relationship between each of the two variables and homoscedasticity assumes that data is equally distributed about the regression line. To compare the *p*‐value against a predefined significance level, one defines the maximum probability of rejecting the null hypothesis when in fact it is true (typically 5% or 1%), the tolerated error or significance level. Pearson's correlation coefficient was considered for *p*‐value < 0.05.

The aim of PCA was to reduce the dimensionality of data. Confounding or even redundant variables were considered by determining Empirical Orthogonal Indices by Principal Components (PCs) with the determination of Empirical Orthogonal Functions. Thus, the first linear combination is the PC that explains most of the variability of the original data set. Similarly, the second PC is orthogonal to the first PC and explains most of the remaining variance of the observation. Data set reduction was achieved by finding linear combinations (PCs) of the original variables that account for as much as possible of the original total variance. The PCA was applied to monthly fire data (Burned_Area), air quality variables (PM10_Obs, PM25_Obs, CO_Obs, O3_Obs, NO2_Obs, WHO_PM10, WHO_PM25, WHO_NO2, AOD_CAMs, BC_AOD_CAMs, and Dust_AOD_CAMs) and meteorological variables (TEMP_Obs, RH_Obs, and WS_Obs) to construct two PCs temporal pollutant‐atmosphere interaction indices called Pollutant‐Burning Interaction (PBI) and Atmospheric‐Pollutant Interaction (API). PCA transformed the actual correlated fire‐pollutant‐meteorological variables into a new set of orthogonal and uncorrelated components. Multicollinearity among multiple variables is resolved by PCA.

The monthly PBI, API, CSD, RSD, PNEU, COPD, and ASMA values were subjected to a Box‐Cox transformation so that the variables resemble a normal distribution. This transformation allows for the application of parametric statistical methods that assume normality, such as constructing confidence intervals and performing hypothesis tests. The PBI and API indices were classified into different groups using *K‐means* cluster analysis. *K‐means* clustering is an unsupervised learning technique that aims to partition data into distinct groups or clusters based on similarity. On this study, *K‐means* cluster analysis was employed to identify temporal patterns in pollutant‐atmosphere interaction represented by the PBI index and API index. To quantitatively assess the quality of the clustering outcomes it was applied *NbClust R Package* (Charrad et al., 2014) to determine the optimal number of clusters. *NbClust* provides 30 different indices that can be used to evaluate the quality of clustering solutions and suggest the best clustering scheme based on various combinations of distance measures, clustering methods, and numbers of clusters (Charrad et al., 2014). In this regard, after performing the *NbClust* and *K‐means* cluster analysis on PBI‐API indices, the data are separated into two groups so that the samples within the same group were as similar as possible and the two different groups (clusters) are as different as possible in their composition. This clustering process helped to identify subpopulations or patterns of association within the data, which can provide valuable insights into the relationships between these fire‐pollutant‐meteorological variables. The identification of distinct groups or patterns through clustering analysis enhanced the understanding of the pollutant‐atmosphere interaction dynamics.

Finally, the two clusters were individually subjected to a Principal Components Linear Regression (PCRL) procedure to examine the statistical relationship between the independent variables (PBI and API) and the dependent variables (CSD, RSD; PNEU; COPD; ASMA). PCRL involves creating new variables (PBI and API indices), called PCs, which are linear combinations of the original independent fire‐pollutant‐meteorological variables. These PCs capture the maximum amount of variance in the data while being uncorrelated with each other. Linear regression is then performed using these PCs (PBI and API) as predictors of the dependent variables (cardio‐respiratory mortality rates). PCRL was applied separately for each dependent variable and the response variable (PBI and API indices) in each cluster aiming to assess the statistical relationships between the fire‐pollutant‐meteorological variables and health outcomes. This approach allows for a more focused analysis within each group and helps uncover specific associations or patterns that may be present. Significant differences in scores between groups were tested at the *p*‐value < 0.05 unless otherwise noted. This threshold is commonly used to assess the strength of evidence in hypothesis testing. Overall, the application of PCRL on each cluster allows for a more granular analysis and can provide insights into the specific associations and patterns between the fire‐pollutant‐meteorological variables and health outcomes in each cluster.

## 3 Results

### 3.1 Burned Area and Air Pollution

**Figure 2:**
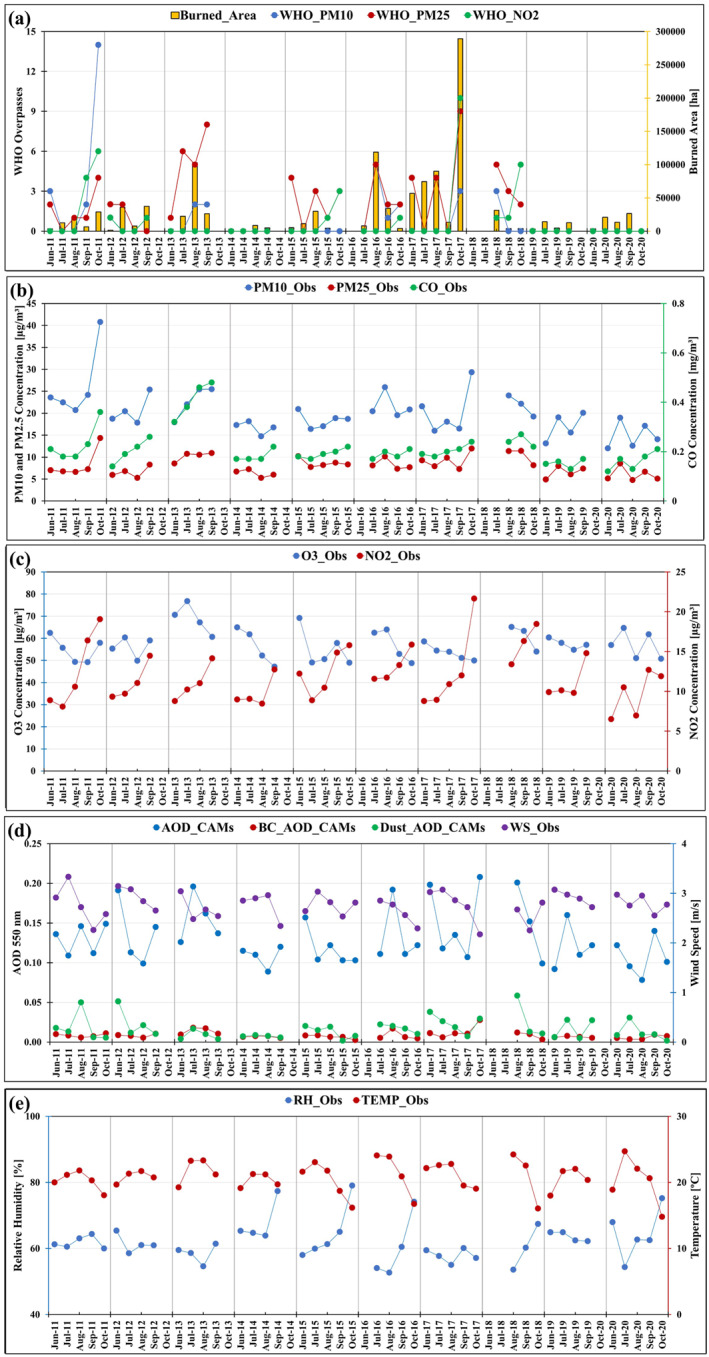
Monthly averages of several variables, from 2011 to 2020, during the fire season (June to October): (a) Burned_Area, WHO_PM10, WHO_PM25, and WHO_NO2 overpasses; (b) PM10_Obs, PM25_Obs, and CO_Obs concentration; (c) O3_Obs and NO2_Obs concentrations; (d) AOD_CAMs, BC_AOD_CAMs, Dust_AOD_CAMs, and WS_Obs; and (e) RH_Obs and TEMP_Obs.

Figure 2a shows monthly Burned_Area and exceedances of WHO_PM10, WHO _PM25, and WHO _NO2 in Portuguese background stations from 2011 to 2020. Figures 2a–2c also illustrates the importance of fires in increasing air pollution concentrations, as the monthly average PM_10_, PM_2.5_, CO, O_3_, and NO_2_ concentrations tend to be higher in the months with larger burned area, such as October 2011, August 2013, August 2016, and October 2017. On the other hand, these months were characterized by favorable meteorological conditions for the development of large wildfires, such as relative humidity below 55% (Figure 2e), high wind speeds (Figure 2d), and the availability of dry vegetation for burning across the country. The different spatial distribution of wildfires together with the different weather conditions, may have contributed to the higher concentrations of PM_10_, PM_2.5,_ NO_2_, and CO in 2011 compared to 2017. Although the PM (particulate matter) shows a high variability for several reasons (variable distance from the air quality monitoring stations in relation to the wildfires, the meteorological conditions for pollution dispersion, size and energy of the wildfires, etc.), this study choose background air quality monitoring stations as a valuable approach to assess the effects of wildfires on air quality as best as possible. Meteorological variables play a crucial role in the formation, dispersion, and transport of atmospheric pollutants, including those generated by wildfires. Wind speed and direction are particularly important factors that greatly affect the dispersion of local and regional transport of pollutants in the atmosphere. Local and regional meteorological conditions can influence how pollutants are transported, the areas they affect, and the duration of exposure for affected populations. Other meteorological factors such as temperature, humidity, and atmospheric stability can also interact with wind speed and direction to further influence the dispersion and behavior of pollutants.

Figure 2d shows AOD_CAMs, BC_AOD_CAMs, and Dust_AOD_CAMs. Although AOD represents the extinction integral of the total atmospheric column due to aerosols, over a given area, it does not directly measure the magnitude of particulate matter concentration, since the particles may be present at different atmospheric levels and not necessarily near the Earth's surface. Nevertheless, the observed air quality (PM_2.5_) impacts were satisfactorily predicted in qualitative terms by the ECMWF CAMS‐Reanalysis. Although AOD is not a direct measure of PM_2.5_ concentrations, it can serve as a proxy or indicator of the total aerosol load in the atmosphere.

### 3.2 Correlation Between Fire‐Pollutants‐Meteorological Components and Mortality

**Figure 3:**
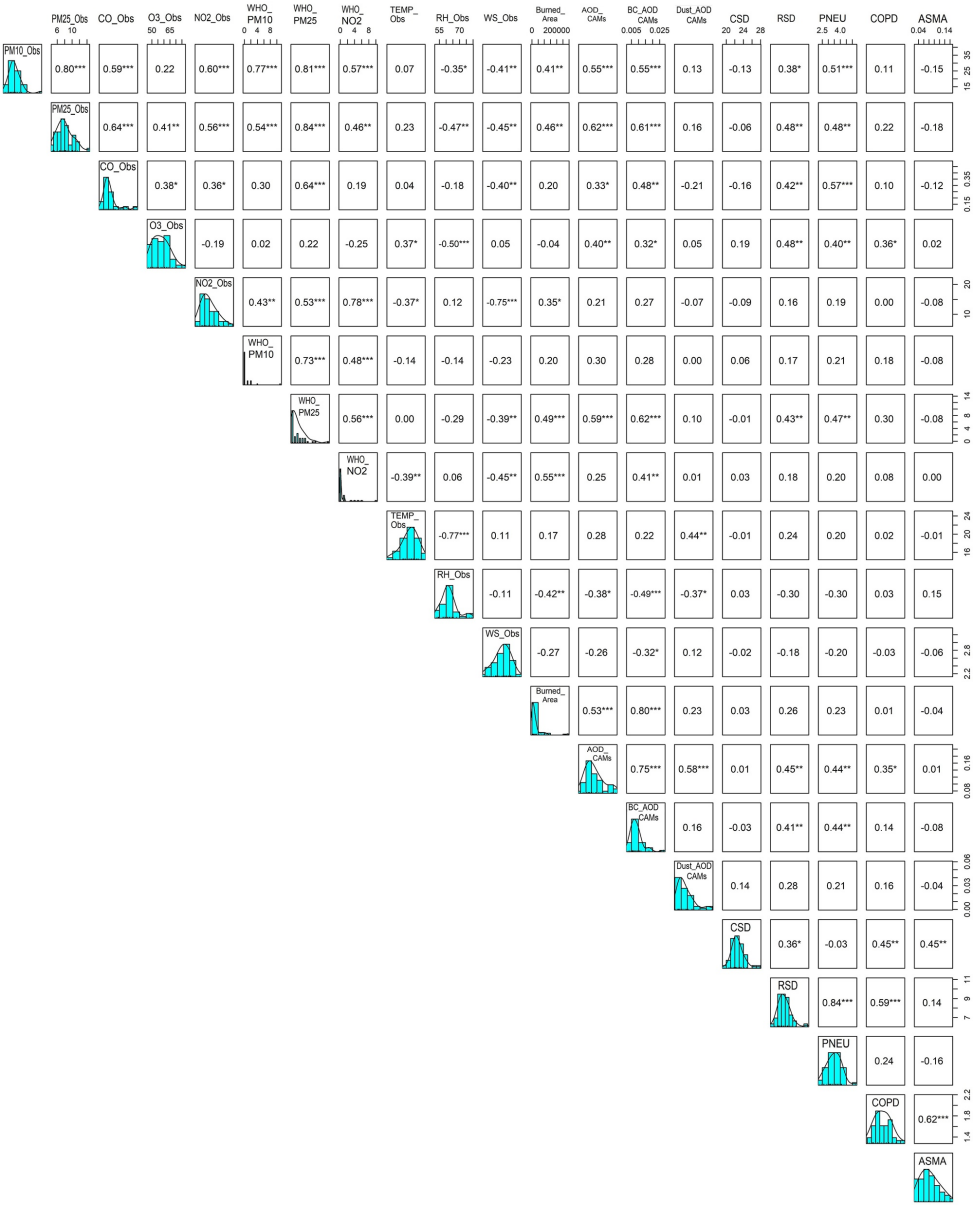
Pairwise correlation matrix between of PM10_Obs, PM2.5_Obs, CO_Obs, O3_Obs, NO2_Obs, WHO_PM10, WHO_PM25, WHO_NO2, TEMP_Obs, RH_Obs, WS_Obs, Burned_Area, AOD_CAMs, BC_AOD_CAMs, Dust_AOD_CAMs, CSD, RSD, PNEU, Chronic Obstructive Pulmonary Disease and ASMA of the fire seasons of 2011–2020: Histograms of each variable are shown along the midline; and the lower panel shows a scatter plot with a linear regression line. Note. *p‐value < 0.05, **p‐value < 0.01, ***p‐value < 0.001.

Figure 3 shows pairwise correlation between of PM10_Obs, PM2.5_Obs, CO_Obs, O3_Obs, NO2_Obs, WHO_PM10, WHO_PM25, WHO_NO2, TEMP_Obs, RH_Obs, WS_Obs, Burned_Area, AOD_CAMs, BC_AOD_CAMs, Dust_AOD_CAMs of the fire seasons of 2011–2020: histograms of each variable are shown along the midline; and the lower panel shows a scatter plot with a linear regression line. Figure 3 provides valuable insight into the correlations between different fire‐pollutant‐meteorological variables and mortality outcomes, as well as the associations between different variables themselves. The direct correlation between fire‐pollutant‐meteorology variables and mortality (CSD, RSD, PNEU, COPD, and ASMA) is shown with statistically significant values marked with asterisks (*). The analysis revealed statistically significant positive correlations between burned area and several variables, including PM_10_ (*r* = 0.41), PM_2.5_ (*r* = 0.46), WHO_PM25 (*r* = 0.49), NO_2_ (*r* = 0.35), AOD (*r* = 0.53) and BC_AOD (*r* = 0.80). These statistically significant correlations indicate that as the burned area increases, there is a corresponding increase in the levels of these pollutants and AOD. Positive correlations among atmospheric pollutants notably PM_10_ showed significant linear correlations with PM_2.5_ (*r* = 0.80) and NO_2_ (*r* = 0.60). This suggests that higher levels of PM_10_ are associated with higher levels of PM_2.5_ and NO_2_.

Statistically significant positive correlations between cardio‐respiratory mortality (CSD, RSD, PNEU, COPD, and ASMA) and several variables, including PM_10_, PM_2.5_, CO, O_3_, WHO_PM25, AOD_CAMs, and BC_AOD_CAMs ranging from 0.38 to 0.59 (Figure 3). In contrast, temperature and relative humidity had a low correlation with CSD, RSD, PNEU, COPD, and ASMA, and not significant (*p*‐value > 0.05) (Figure 3). The absence of a significant association between temperature/relative humidity and health outcomes does not necessarily mean that these variables do not play a role in overall mortality. Other factors such as air pollution, socioeconomic factors, population density, and individual health behaviors may confound the relationship between temperature/relative humidity and health outcomes.

The analysis of Table 1 shows the value of KMO and Bartlett's test of sphericity determine if fire‐pollutant‐meteorological data are appropriate for the multivariate statistical analysis. Kaiser‐Meyer Olkin sampling adequacy was 0.66 and the Bartlett's test of sphericity was found to be 0.000 which means highly significant (Table 1). A KMO value over 0.5 and a significance level for the Bartlett's test below 0.000 suggest there is substantial correlation in the data and is adequate for multivariate statistical analysis. The KMO Bartlett's test relates to the importance of the study, thereby shows the reliability and validity.

**Table 1:** Kaiser‐Meyer Olkin and Bartlett's Test

KMO and Bartlett's test		
Kaiser‐Meyer‐Olkin measure of sampling adequacy		0.66
Bartlett's test of sphericity	Approx. Chi‐Square	10,530.32
	Df	105
	*p*‐value	0.000

According to Figure 3, high correlations were detected between PM10_ObsxPM2.5_Obs (*r* = 0.80), PM10_ObsxWHO_PM25 (*r* = 0.81), PM2.5_ObsxWHO_PM25 (*r* = 0.84), Burned_AreaxBC_AOD_CAMs (*r* = 0.80). To evaluate the existence of collinearity between variables highly correlated (Pearson *r* ≥ 0.80) it was used TI and VIF with cut‐off points of more than 0.1 and not exceeding 10 respectively. The result from Table 2 shows that the tolerance values range from 0.29 to 0.44 significantly higher than 0.10. Also, the VIF ranges between 2 and 3, which is less than 10, thus concluded that the problem of multicollinearity does not exist among the independent variables. A VIF value between 1 and 5 indicates moderate correlation between two variables, but this is often not severe enough to require attention. From table 2, it can be concluded that there is no serious multi‐collinearity or confounding between the independent variables.

**Table 2:** Tolerance (TI) and Variance Inflation Factor (VIF) Values for the Highly Correlated Independent Variables

Variables	Collinearity statistics		
	PM10_Obs	PM25_Obs	WHO_PM25
	TI	TI	TI
PM10_Obs	–	0.34	0.36
PM25_Obs	0.29	–	0.36
WHO_PM25	0.29	0.34	–

	PM10	PM25_Obs	WHO_PM25
	VIF	VIF	VIF
PM10_Obs	–	3	3
PM25_Obs	3	–	3
WHO_PM25	3	3	–

	BC_AOD_CAMs	
	TI	
Burned_Area	0.44	

	BC_AOD_CAMs	
	VIF	
Burned_Area	2	

**Figure 4:**
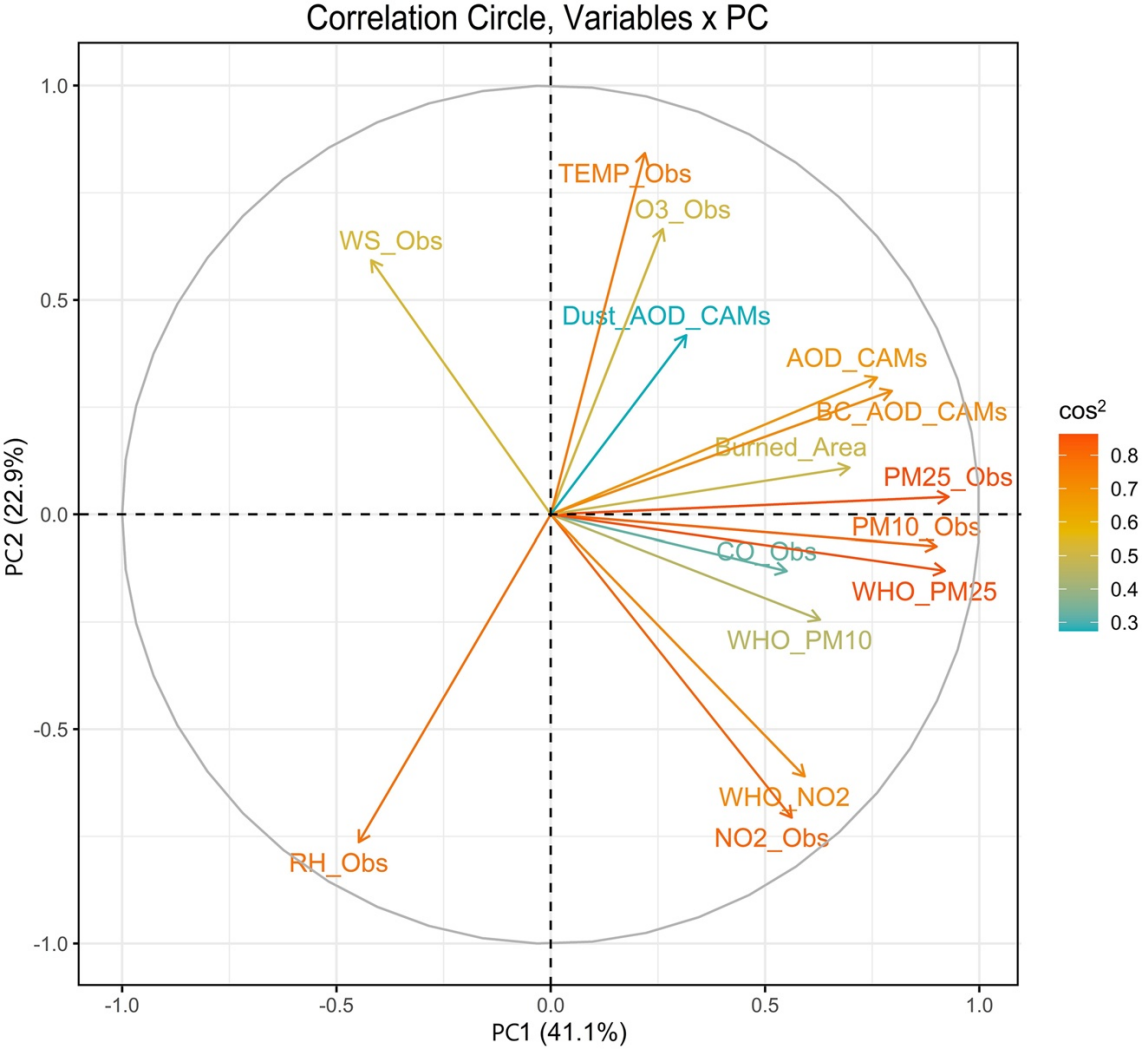
Correlation circle through Principal Component Analysis for monthly data. Vectors indicate the strength and direction of each variable fire‐pollutant‐meteorology to PC1 and PC2. The cos^2^ represents the quality of the variables' representation on the factor map.

By applying PCA to reduce the dimensionality of the data and constructing temporal pollutant‐atmosphere interaction indices, it was possible to capture the relative contribution of each variable to the PCs and correlate the with health outcome. The PCs are linear combinations of the fire‐pollutant‐meteorological data preserving as much of the variance as possible. This approach helped capture the relative contributions of each variable to the PCs. The results of the PCA are shown in Table 3 and Figure 4. These results show the explained variance resulting from the fire‐pollutant‐meteorology variable data. According to the criterion of the percentage of explained variance, the first two PCs explain more than 62% of the variance in the data set. In the PC1 composition, PM25_Obs (13%), PM10_Obs (13%), WHO_PM25 (13%), BC_AOD_CAMs (10%), AOD_CAMs (7%), Burned_Area (7%), WHO_NO2 (6%), NO2_Obs (6%), CO_Obs (6%), and WHO_PM10 (6%), make the largest contribution as shown by the results in Table 3. The percentage of explained variance indicates the amount of information each PC captures from the data set. These variables account for more than 90% of the total explained variance in PC1, suggesting that they capture the most significant patterns in the data. PC1 is strongly correlated with air pollutants and wildfires during the fire season in Portugal, indicating their dominant influence on this component.

**Table 3:** Correlations Between the Original Variables and the First Two Principal Components (PCs) and the Contributions of Each Variable to the PCs

Variable	Correlation between variables × PC		Contribution of the variables (%)	
	PC1	PC2	PC1	PC2
PM10_Obs	0.886	−0.090	12.59	0.27
PM25_Obs	0.903	0.094	13.10	0.29
CO_Obs	0.640	−0.042	6.56	0.06
O3_Obs	0.287	0.580	1.32	11.01
NO2_Obs	0.639	−0.626	6.55	12.83
WHO_PM10	0.639	−0.254	6.55	2.11
WHO_PM25	0.901	−0.092	13.03	0.28
WHO_NO2	0.633	−0.550	6.43	9.90
TEMP_Obs	0.133	0.845	0.28	23.40
RH_Obs	−0.436	−0.749	3.06	18.36
WS_Obs	−0.538	0.427	4.64	5.96
Burned_Area	0.658	0.105	6.95	0.36
AOD_CAMs	0.722	0.377	8.36	4.64
BC_AOD_CAMs	0.786	0.249	9.93	2.02
Dust_AOD_CAMs	0.200	0.511	0.64	8.53

For PC2, Table 3 PC2 is mainly influenced by variables such as TEMP_Obs (23%), RH_Obs (18%), NO2_Obs (13%), O3_Obs (11%), WHO_NO2 (13%), Dust_AOD_CAMs (9%), and WS_Obs (6%). These variables contribute more than 90% to the explained variance in PC2. PC2 is strongly correlated with temperature, relative humidity, and ozone concentration near the surface. PC2 was strongly correlated with temperature, relative humidity, ozone concentration near the surface. The negative correlation of NO_2_ in PC2 indicate complex behavior of O_3_ during the fire season. The PC2 shows the complex behavior between O_3_ and NO_2_. The results indicate that the temperature value and the presence of NO_2_ influence the O_3_ concentration. O_3_ is formed by a series of chemical reactions, under the influence of sunlight, involving VOCs combined with a group of air pollutants known as nitrogen oxides (NO_*x*_). The results of the PCA provide valuable insights into the key variables driving the patterns in the fire‐pollutant‐meteorology data set. PC1 primarily represents the influence of air pollutants and wildfires, while PC2 captures the interplay between temperature, relative humidity, and ozone concentration.

Figure 4 shows the evaluation of each variable contribution for PC1 and PC2. The representation quality of the variables on the factor map is referred to as cos^2^ (squared cosine, squared coordinates). A high cos^2^ value indicates a good representation of the variable on the PC, while a low cos^2^ value indicates that the variable is not perfectly represented by the PCs. The closer a variable is to the correlation circle, the better its representation on the factor map. The gradient colors of the cos^2^ also indicate good or poor representation of the variable in the correlation circle. Figure 4 shows that the fire (Burned_Area) and pollutant variables (PM25_Obs, WHO_PM25, PM10_Obs, BC_AOD_CAMs, AOD_CAMs, Burned_Area, WHO_NO2, NO2_Obs, CO_Obs, and WHO_PM10) are highly correlated variables and strongly correlated with the PC1 (represented by the horizontal axis; *p*‐value < 0.05). In PC2, the variables with the highest correlation and statistically significant (*p*‐value < 0.05) are TEMP_Obs, RH_Obs, NO2_Obs, O3_Obs, WHO_NO2, Dust_AOD_CAMs, and WS_Obs. Overall, Figure 4 confirms the strong correlations between the fire‐pollutant‐meteorology variables and the PCs (PC1 and PC2). The variables associated with fire and pollutants are highly correlated with PC1, while the variables related to temperature, humidity, NO_2_, O_3_, and wind speed are strongly correlated with PC2.

Thus, PC1 and PC2 are the PCs that best represent the data distribution, and the *scores* are the projections of the data onto the PCs. In this sense, PC1 and PC2 *scores* are used as two pollutant‐atmosphere interaction indices. The PC1 *score* represents the PBI index because it reflects the strong correlation between pollutants and burned area, which contribute most to the formation of PC1. The PBI index captures the interaction between pollutants and the occurrence of fires, highlighting the combined effects of these variables. On the other hand, the PC2 *score* represents the Atmosphere‐Pollutant Interaction (API) index. In the formation of API, variables such as temperature, relative humidity, ozone, and dust contributed more significantly than the pollutants. This index, API, highlights the interaction between these atmospheric variables and pollutants. The novels independent variables (PBI and API) successively maximize variance.

**Figure 5:**
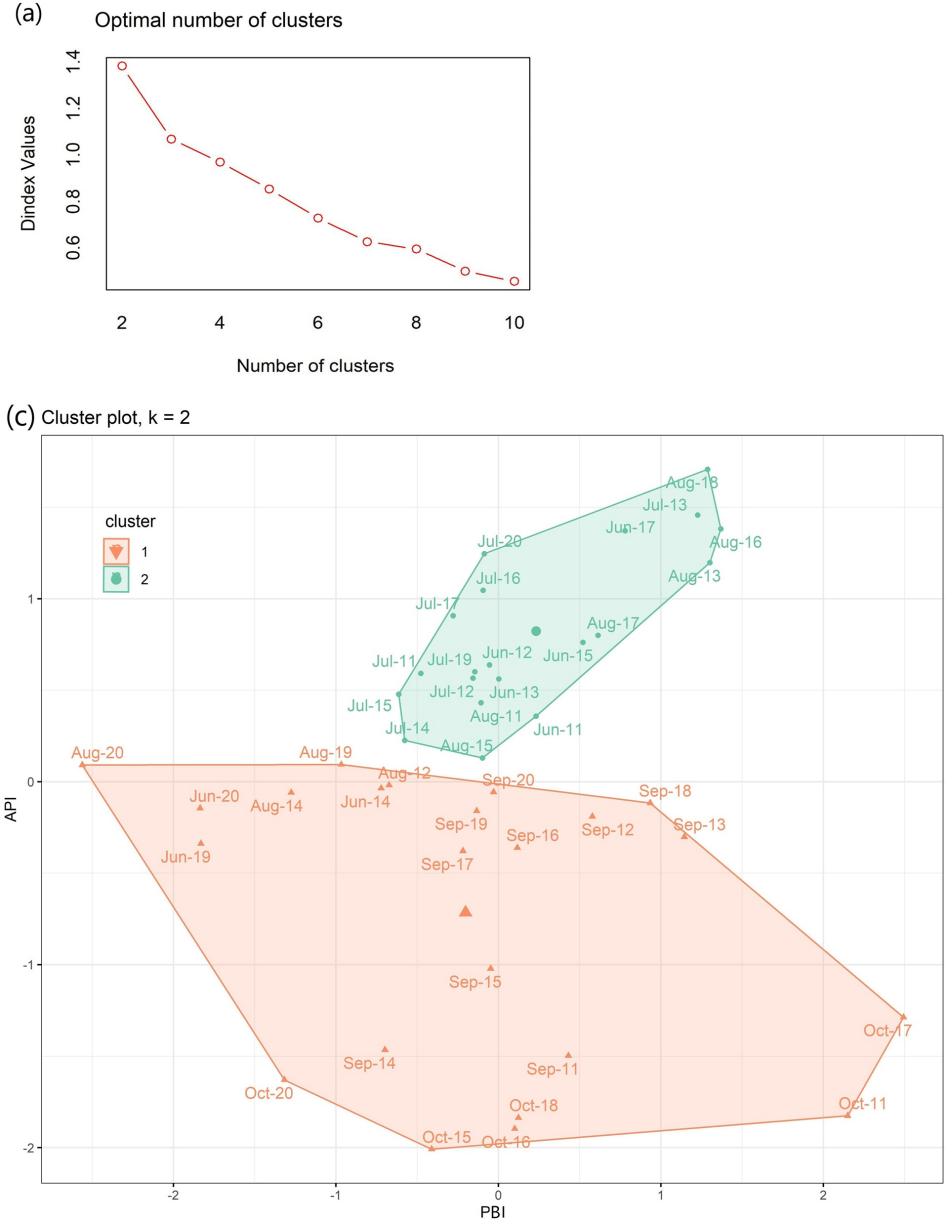
(a) Plot of the Dindex graphic for determining the best number of clusters in the simulated data set using *NbClust* R package. Dindex proposes 2 as the optimal number of clusters, *k* = 2; (b) *k‐means* clustering result for the PBI‐API indices. The plots refer to the PBI‐API indices.

PBI and API indices were submitted to cluster analysis to identify temporal patterns in pollutant‐atmosphere interaction. Figures 5a and 5b shows optimal number of clusters through *NbClust* (Figure 5a) and the clusters distribution through *K‐means* cluster analysis (Figure 5b) applied to PBI‐API indices. The *NbClust* R package is a useful tool for determining the optimal number of clusters in a data set. By applying the *NbClust* package and considering the various indices and clustering options, it was determined that the data set is best represented by two clusters (Figure 5a), indicating the presence of distinct patterns or subpopulations within the data. By examining the clusters and their characteristics, valuable insights were gained regarding the relationships between fire‐pollutants‐meteorological variables (Figure 5b). Figure 5b provides clustering results yielded by *K‐means*. *K‐Means* cluster analysis applied to the PBI‐API indices divided the data set into two clusters, Cluster 1, and Cluster 2 (Figure 5b).

**Figure 6:**
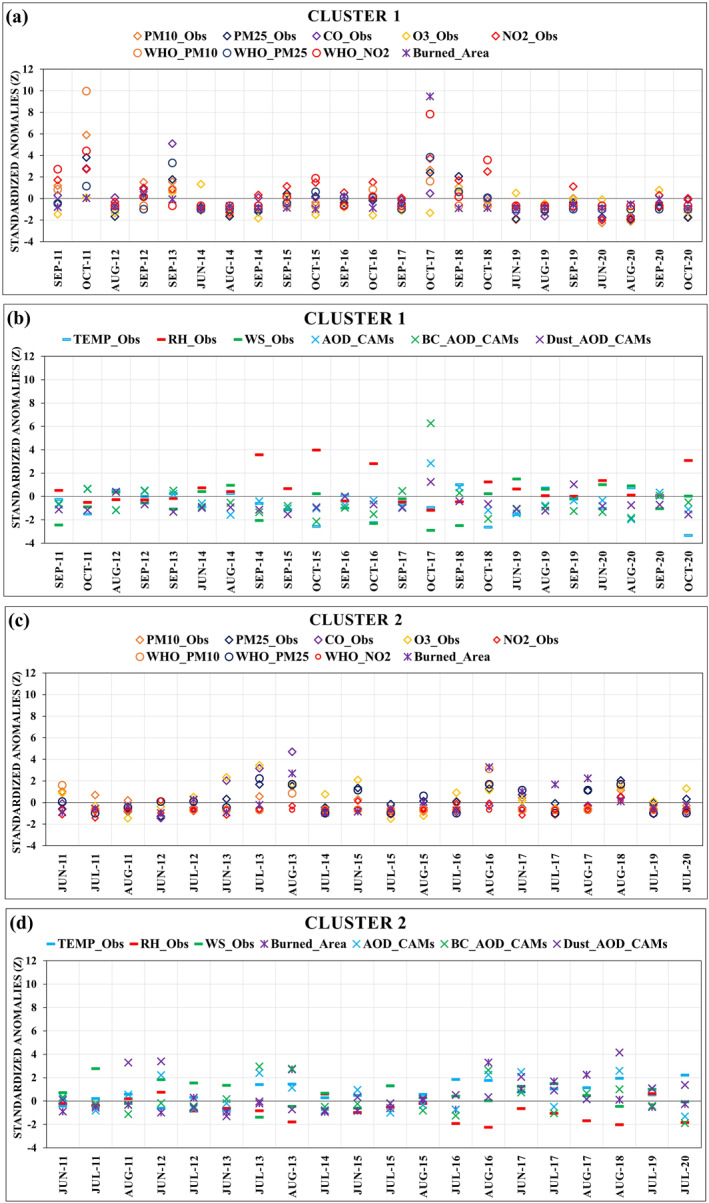
Variability of standardized anomalies (*Z‐scores*) of the variables PM10_Obs, PM2.5_Obs, CO_Obs, O3_Obs, NO2_Obs, WHO_PM10, WHO_PM25, WHO_NO2, TEMP_Obs, RH_Obs, WS_Obs, Burned_Area, AOD_CAMs, BC_AOD_CAMs, Dust_AOD_CAMs of the fire seasons of 2011–2020: (a, b) Cluster 1 and (c, d) Cluster 2.

The standardized fire‐pollutant‐meteorological data were monthly spread according to Cluster 1 and Cluster 2 (Figures 6a–6d). Cluster 1 (Figures 6a and 6b) includes the months inside the wildfire seasons with lower temperature, higher relative humidity, and higher NO_2_ concentration near the surface. Cluster 2 (Figures 6c and 6d), focuses mainly in the months of June, July, and August. These months represent summer in Europe and include the periods with the most extreme weather conditions, such as higher temperatures, lower relative humidity, larger forest fires, higher PM_10_, PM_2.5_, and O_3_ concentrations near the surface, and high AOD. WHO_PM10 were higher in Cluster 1 than in Cluster 2 while WHO_PM25 were higher in Cluster 2 than Cluster 1 showing the importance of keeping these variables in the analysis.

Finally, the two clusters were subjected to PCs linear regression analysis to better understand the relationship between health outcomes (CSD, RSD, PNEU, COPD, and ASMA) and PBI and API indices (PBI and API). From 2011 to 2020, the monthly average number of deaths due to cardio‐respiratory diseases (CSD, RSD, PNEU, COPD, ASMA) during the fire season in Portugal (June to October) was 7.15 (±0.5) deaths per hundred thousand habitant and per month (Dth hd^−1^ mh^−1^). From 2011 to 2020, the mean number of deaths due to CSD was 22.67 (±1.0) Dth hd^−1^ mh^−1^, while the number of deaths due to RSD was 7.86 (±0.6) Dth hd^−1^ mh^−1^, due to PNEU was 3.46 (±0.5) Dth hd^−1^ mh^−1^, due to COPD was 1.66 (±0.2) Dth hd^−1^ mh^−1^ and due to ASMA was 0.08 (±0.03) Dth hd^−1^ mh^−1^.

**Figure 7:**
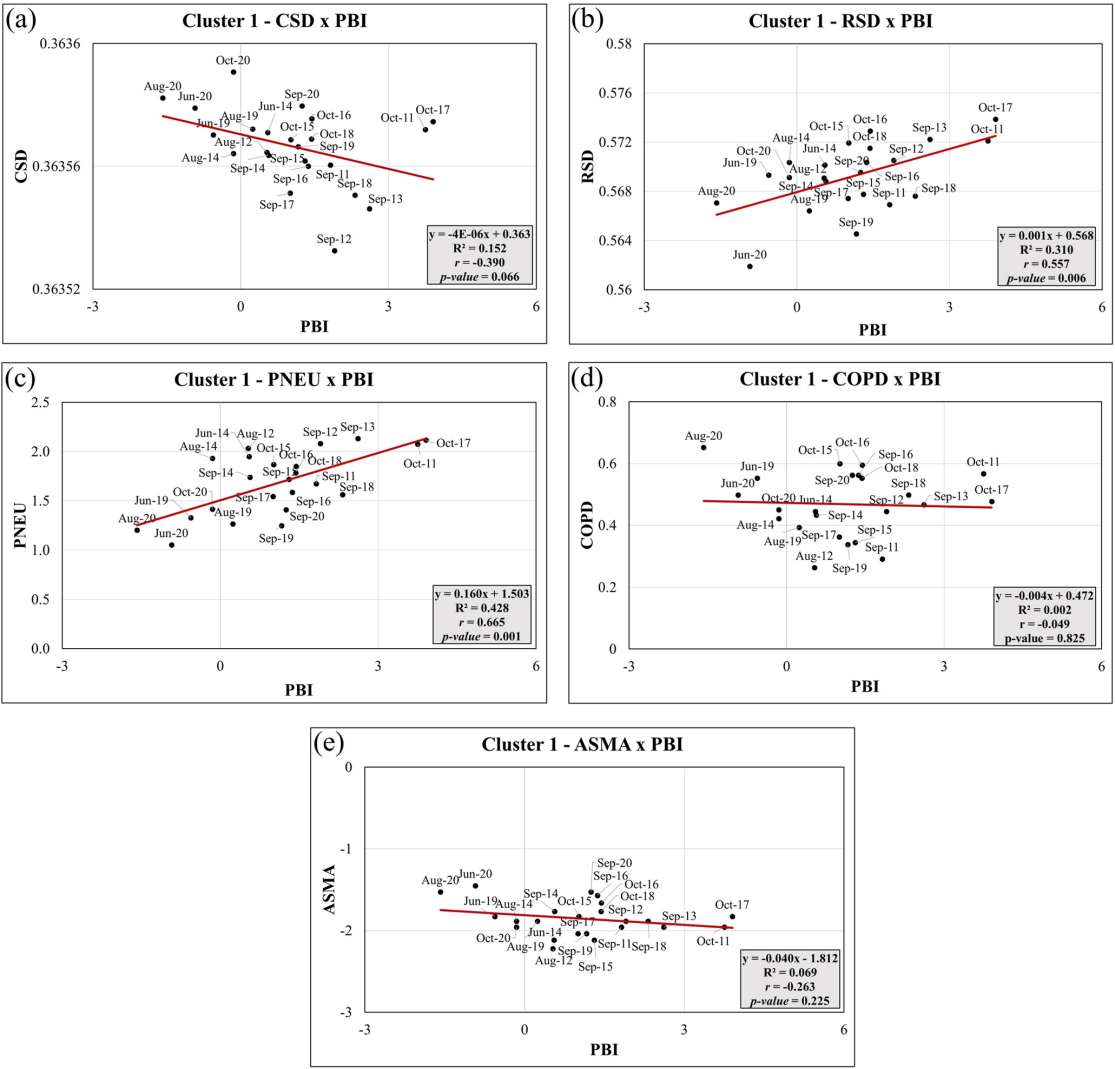
Principal Components Linear Regression analysis between health outcomes and Pollutant‐Burning Interaction (PBI) index for Cluster 1: (a) CSDxPBI; (b) RSDxPBI; (c) PNEUxPBI; (d) COPDxPBI; (e) ASMAxPBI.

Figures 7a–7e shows the correlation between the different health outcomes and PBI index for Cluster 1. A strong statistically significant (*p*‐value < 0.05) positive correlation was found between RSDxPBI (*r*_RSD_ = 0.58) and PNEUxPBI (*r*_PNEU_ = 0.67) (Figures 7b–7c), while no statistically significant correlation was found between CSDxPBI, COPDxPBI, and ASMAxPBI (Figures 7a, 7d and 7e). Figure 7 also show *R‐squared* (*R* ^2^) which indicates the variance in the dependent variable that the independent variables explain collectively which is basically measure of the strength of the relationship between the model and the dependent variable. For RSDxPBI, the *R* ^2^ value was 0.31, indicating that 31% of the variance in RSD can be explained by the PBI index. Similarly, for PNEUxPBI *R* ^2^ the R2 value was 0.43, suggesting that 43% of the variance in PNEU can be explained by the PBI index. According to the PCA analyses, PBI index is highly correlated with the atmospheric pollutants PM_2.5_, PM_10_, CO, and NO_2_ concentrations near the surface, as well as with WHO_PM10, WHO_PM25, and WHO_NO2 exceedances of the WHO guidelines during the 2011–2020 fire seasons. PBI were also correlated with Burned_Area, BC_AOD_CAMs, AOD_CAMs. As Cluster 1 mainly includes the months within the fire season with lower temperatures and higher relative humidity, the statistically significant positive correlation between PBI and mortality due to RSD and PNEU may be associated to the high concentration of pollutants near the surface, especially higher NO_2_ and PM_10_, and the burned area. These factors, combined with the specific atmospheric conditions during the fire season, may contribute to the observed correlations between PBI and health outcomes.

**Figure 8:**
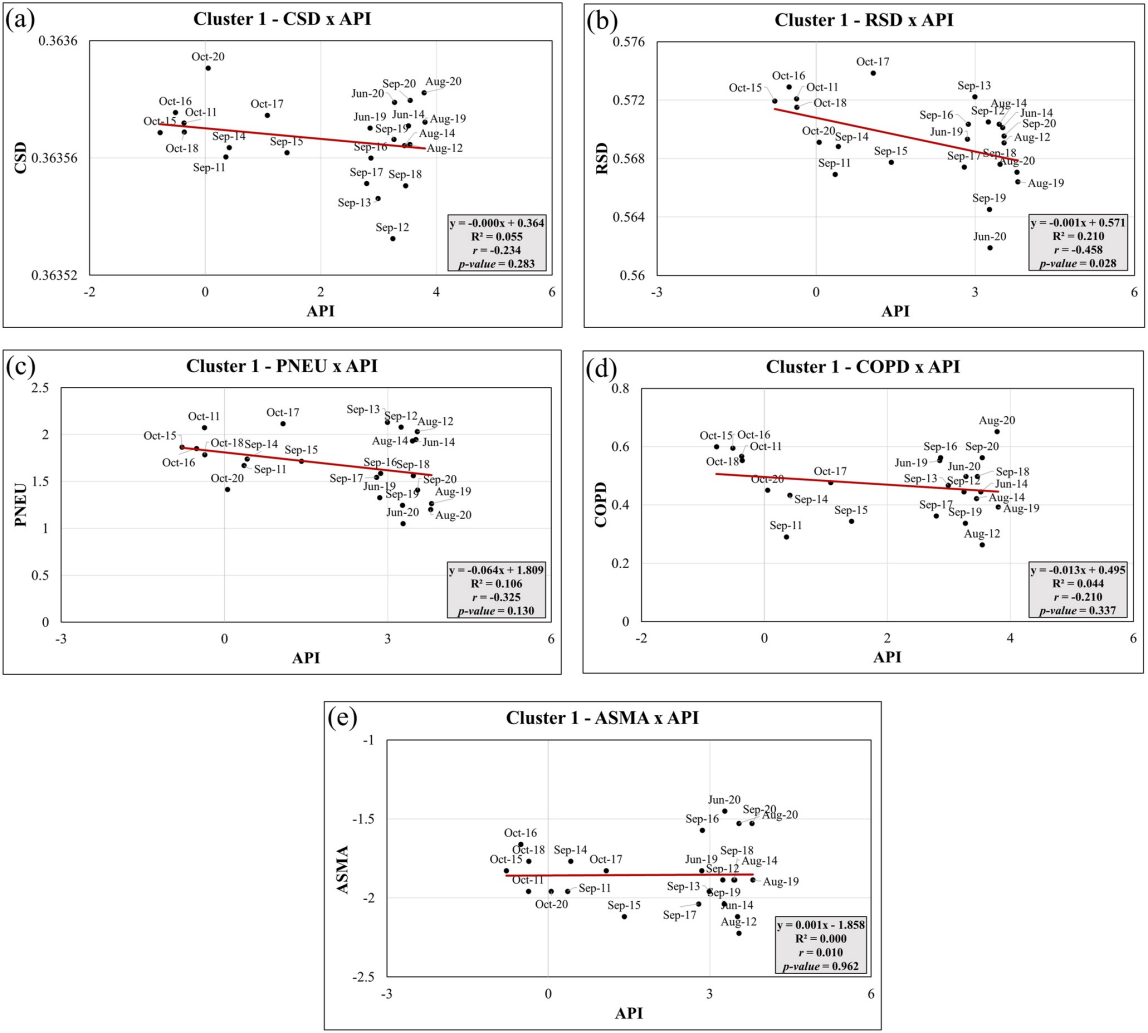
Principal Components Linear Regression analysis between health outcomes and Atmospheric‐Pollutant Interaction (API) index for Cluster 1: (a) CSDxAPI, (b) RSDxAPI; (c) PNEUxAPI; (d) COPDxAPI; (e) ASMAxAPI.

Figures 8a–8e shows the correlation between the different health outcomes considered and API index for Cluster 1. In this case, the correlations between mortality causes and API index (CSDxAPI, RSDxAPI, PNEUxAPI, COPDxAPI, and ASMAxAPI) do not show statistically significance (*p*‐value > 0.05). Atmospheric‐Pollutant Interaction was most strongly related to temperature, relative humidity and O_3_. However, since Cluster 1 included mostly colder and wetter months, the API index in Cluster 1 were not related to extreme weather conditions. This suggests that during the wildfire season, colder and wetter months with cleaner air were not associated with an increase or decrease in cardio‐respiratory mortality rates. These findings indicate that, within Cluster 1, the API index did not exhibit a significant relationship with the health outcomes considered. Other factors beyond atmospheric pollutants, such as temperature, relative humidity, and O_3_, may play a more prominent role in influencing mortality rates during the specific conditions of Cluster 1, characterized by colder and wetter months.

**Figure 9:**
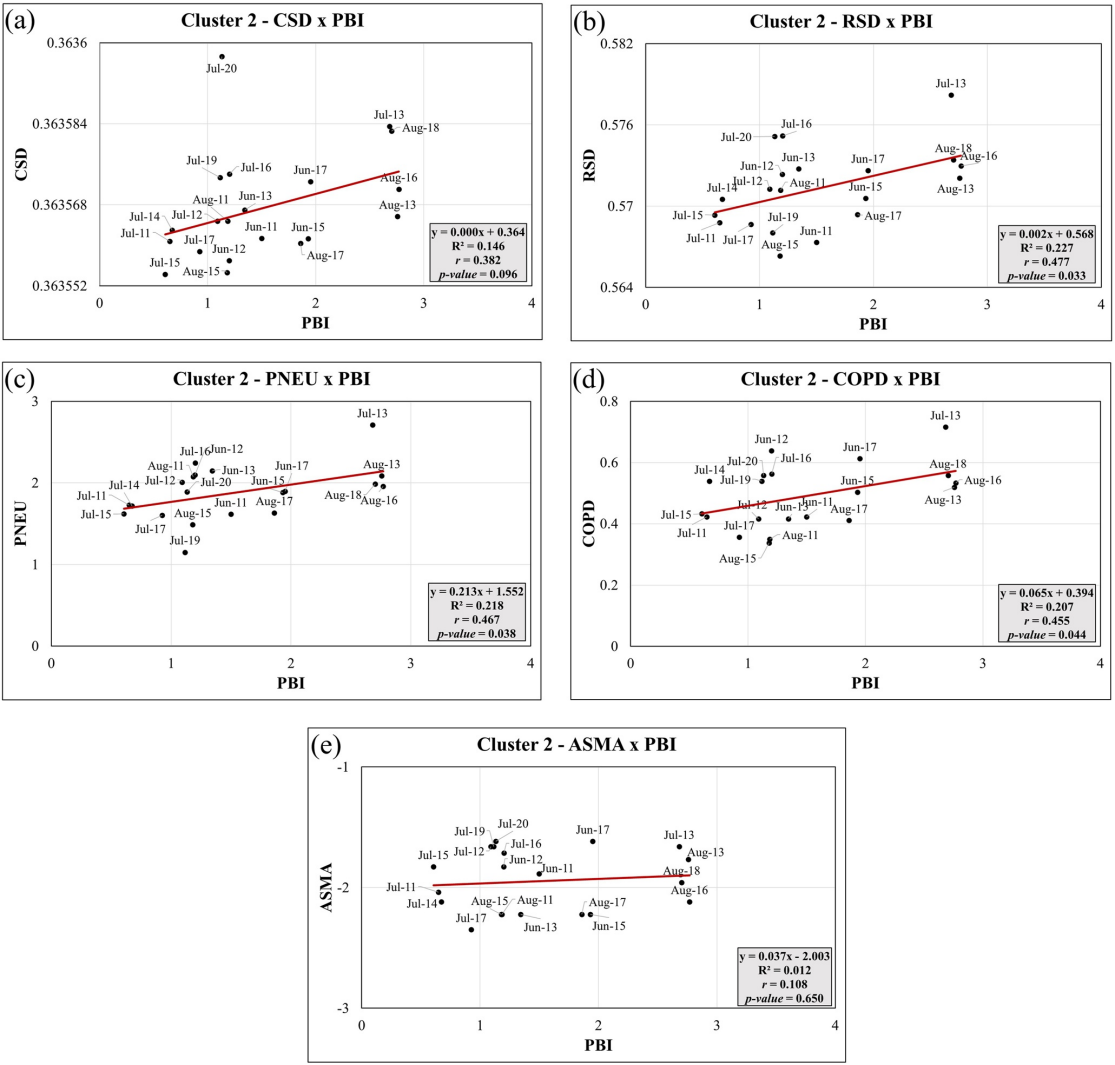
Principal Components Linear Regression analysis between health outcomes and Pollutant‐Burning Interaction (PBI) index (PBI) for Cluster 2: (a) CSDxPBI, (b) RSDxPBI; (c) PNEUxPBI; (d) COPDxPBI; (e) ASMAxPBI.

Cluster 2 primarily consists of the warmest and driest months of the year, namely June, July, and August, which also coincide with the peak wildfire season. These months are characterized by more extreme weather conditions, including higher temperatures, lower relative humidity, larger forest fires, and higher concentrations of PM_10_, PM_2.5_, and O_3_ near the surface, as well as elevated AOD levels. Figure 9b–9d show statistically significant (*p*‐value < 0.05) positive correlations between RSDxPBI (*r*_RSD_ = 0.48), PNEUxPBI (*r*_PNEU_ = 0.47) and COPDxPBI (*r*_COPD_ = 0.45). Regarding to Cluster 2, PBI were positively associated with RSD, PNEU, and COPD. These correlations suggest that RSD, PNEU, and COPD deaths may be influenced by a combination of higher pollutant concentrations such as PM_10_, PM_2.5_, CO, and NO_2_, along with the occurrence of larger wildfires. The *R* ^2^ values for RSDxPBI, PNEUxPBI, and COPDxPBI indicate that the variations in these health outcomes explained collectively by the PBI index range from 20% and above. Figures 9a and 9e shows not statistically significant (*p*‐value > 0.05) relationship between CSDxPBI (*r*_CSD_ = 0.38) and ASMAxPBI (*r*_ASMA_ = 0.11), although the correlation was positive. Nonetheless, linear regression used as a diagnostic method to identify cause‐of‐death patterns during the fire season, suggests that deaths due to CSD and ASMA also tend to increase due to extreme atmospheric conditions associated with fire‐pollutant meteorology components. Overall, in Cluster 2, the PBI index demonstrates positive associations with RSD, PNEU, and COPD, indicating that the combination of higher pollutant concentrations and larger wildfires can impact mortality rates related to these respiratory conditions.

**Figure 10:**
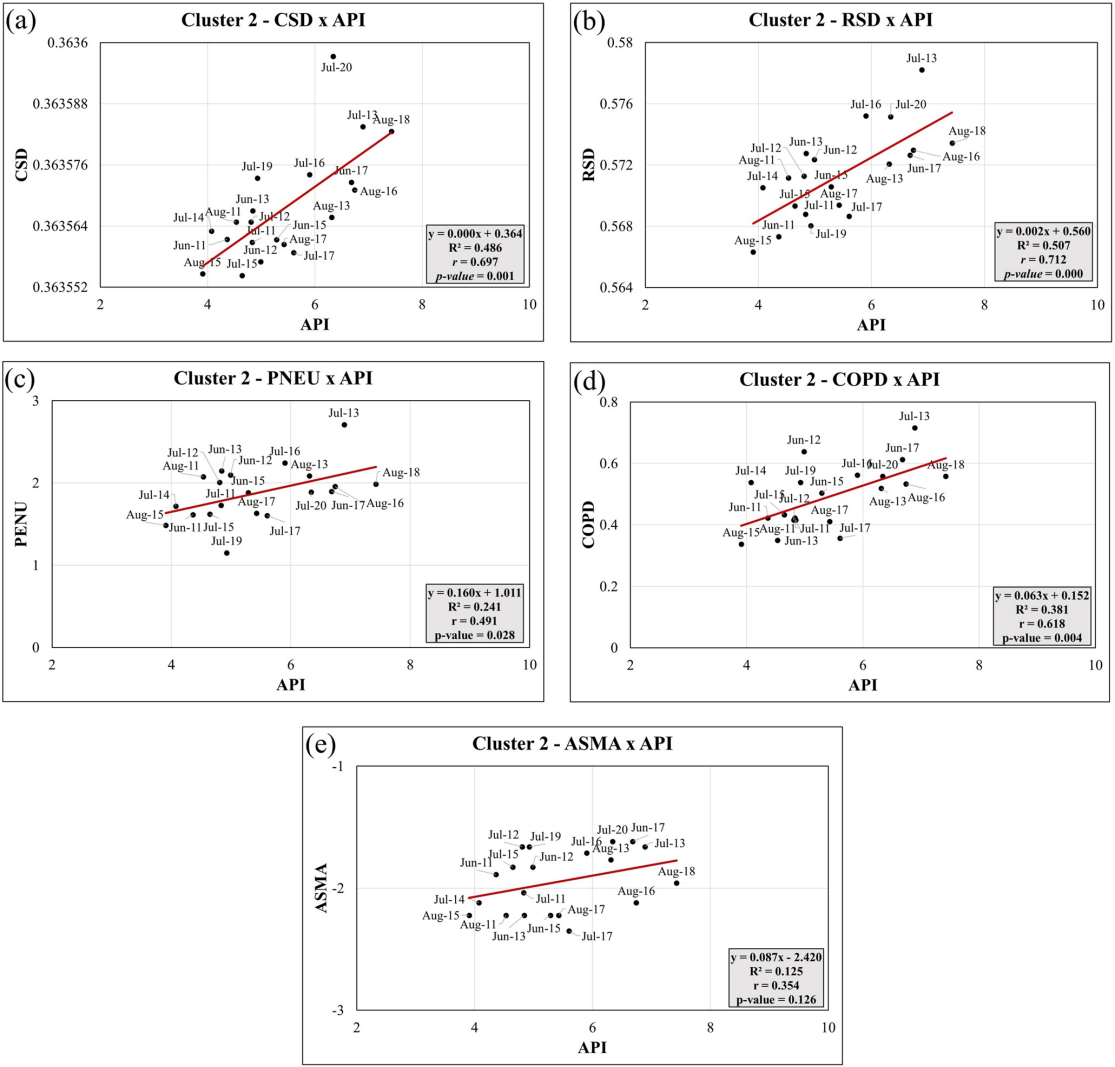
Principal Components Linear Regression analysis between health outcomes and Atmospheric‐Pollutant Interaction (API) index for Cluster 2: (a) CSDxAPI, (b) RSDxAPI; (c) PNEUxAPI; (d) COPDxAPI; (e) ASMAxAPI.

Figures 10a–10d shows statistically significant (*p*‐value < 0.05) correlations in Cluster 2 between CSDxAPI (*r*_CSD_ = 0.70), RSDxAPI (*r*_RSD_ = 0.71), PNEUxAPI (*r*_PNEU_ = 0.49), and COPDxAPI (*r*_COPD_ = 0.62). These correlations suggest that the API index, which represents extreme weather conditions characterized by high temperature, low relative humidity, and high near‐surface O_3_ concentration, is strongly correlated with CSD, RSD, and COPD (with correlation coefficients above 0.6). The *R*‐squared (*R* ^2^) values for CSDxAPI and RSDxAPI indicate that the API index explains more than 50% of the variations in these health outcomes, while for PNEUxAPI and COPDxAPI, the API index explains more than 25% of the variations. Figure 10e shows not statistically significant (*p*‐value > 0.05) correlations in Cluster 2 between ASMAxAPI (*r*_ASMA_ = 0.364). However, the correlation was positive suggesting that ASMA‐related deaths also tend to occur more frequently in these months.

## 4 Discussion

In general, several time‐series studies have examined the relationship between adverse health outcomes and air pollution as well as meteorological variables (Augusto et al., 2020; Jurado et al., 2020; Pacheco et al., 2020; Tarín‐Carrasco et al., 2021). Long‐term exposure to NO_2_, which is a toxic gas and a primary pollutant precursor of O_3_ in the troposphere (Andino‐Enriquez et al., 2018; Bortoli et al., 2009), is associated with hypertension, pulmonary dysfunction, and COPD (Lamichhane et al., 2018; Lyons et al., 2020). NO_2_ also increases the risk of developing viral infections (Jurado et al., 2020; Pacheco et al., 2020). Augusto et al. (2020) showed that PM_10_ released during the October 2017 megafires in Portugal had a significant impact on natural and cardio‐respiratory mortality on smoky days. For each additional 10 μg/m^3^ of PM_10_, there was a 0.89% increase (95% confidence interval, 0%–1.77%) in the number of natural deaths and a 2.34% increase (95% confidence interval, 0.99%–3.66%) in the number of cardio‐respiratory deaths (Augusto et al., 2020). By using Poisson regression models, Tarín‐Carrasco et al. (2021) found a significant positive correlation between burned area and PM10 in some regions of Portugal, as well as a significant association between PM10 concentrations and all‐cause (excluding injuries, poisoning and external causes) and cause‐specific mortality (circulatory and respiratory).

In this work, PPCA was applied to fire‐pollutant‐meteorological variables to reduce the dimensionality of the data and successively maximize the variance of the data. The first two PCs (PC1 and PC2) (accounting for more than 62% of the total variance in the data set) were used as two pollutant‐atmosphere interaction indices, named PBI and API. The PBI and API indices were determined by constructing PCs based on atmospheric and pollutant variables. PBI was strongly correlated with air pollutants and burned area, while API was strongly correlated with meteorological variables such as air temperature and relative humidity, and O_3_. The PBI‐API indices were subjected to *K‐means* cluster analysis to cluster the data into an optimal number of clusters. By applying *K‐means* clustering to these indices, the data points (representing different time periods) were grouped into clusters based on their similarity in terms of pollutant‐atmosphere interaction patterns. Each cluster contains data points that have similar patterns of association between fire, pollutants, and meteorological variables. Two clusters were created from the PBI‐API indices, labeled Cluster 1 and Cluster 2. Cluster 1 includes the months within the wildfire season with lower temperature, higher relative humidity, higher near‐surface NO_2_ concentration, and higher WHO_PM10. Cluster 2 includes the months with the most extreme weather conditions, such as higher temperature, lower relative humidity, larger wildfires, higher PM_10_, PM_2.5_, and O_3_ concentrations near the surface, and high AOD.

Once the PBI and API indices were determined, their dependence on five cardio‐respiratory disease was measured through the determination and accuracy of the fit of a linear regression model. Therefore, to assess how these two PBI‐API indices correlated with cardio‐respiratory mortality rates due to CSD, RSD, PNEU, COPD and ASMA during the 2011–2020 wildfire seasons in Portugal, the two clusters were subjected to PCs linear regression analysis. The results showed a statistically significant (*p*‐value < 0.05) correlation between fire‐pollutant‐meteorological indices and cardio‐respiratory mortality during the wildfire seasons in Portugal, specifically CSD, RSD, PNEU, and COPD. If the linear regression model shows a significant association between the PBI and API indices and cardio‐respiratory diseases, it indicates that these indices can serve as indicators or predictors of disease outcomes. The accuracy of the fit of the model helps evaluate how well the PBI and API indices explain the variation in the diseases of interest.

It is now widely acknowledged that air pollution consists of complex mixtures of pollutants, and that individuals are often exposed to multiple pollutants simultaneously (Cao et al., 2021; Dominici et al., 2002; Moolgavkar, 2003; Roberts & Martin, 2006). Assessing the health effects of air pollution mixtures and atmospheric variables combined it allows for a more realistic representation of the exposure scenario, considering the complex and diverse nature of air pollution. Second, it provides a better understanding of the combined effects of pollutants that may not be apparent when they are studied in isolation (Cao et al., 2021; Roberts & Martin, 2006). Third, it enables policymakers to identify specific pollutant mixtures that have the greatest impact on public health, thereby facilitating targeted interventions and mitigation strategies.

Cao et al. (2021) introduced two methods for estimating the joint effects of multiple pollutants: the cumulative risk index (CRI) and supervised principal component analysis (SPCA). These methods aimed to provide a more accurate assessment of the combined health risks associated with multiple pollutants. By constructing three types of Air Quality Health Index (AQHIs) based on the standard method, CRI and SPCA, Cao et al. (2021) compared their validity and performance as communication tools. The evaluation included examining their relationship with mortality and assessing their ability to capture the overall health risk of pollution mixtures, particularly concerning cause‐specific mortalities (Cao et al., 2021). The results of their study demonstrated that all three AQHIs, including the standard method, CRI‐AQHI, and SPCA‐AQHI, exhibited a linear relationship with mortality. This suggests that all three indices are effective in conveying the health risks associated with air pollution (Cao et al., 2021).

Our study found statistically significant positive correlations in Cluster 1 between RSDxPBI and PNEUxPBI, *r* > 0.50 while no statistically significant correlations were found between cardio‐respiratory mortality rates and API index in Cluster 1 indicating that months within the wildfire season with lower temperature and higher relative humidity, were not associated with an increase or decrease in cardio‐respiratory mortality rates. Cluster 2 showed statistically significant positive correlations between RSDxPBI, PNEUxPBI, and COPDxPBI, *r* > 0.40. Regarding the API index, Cluster 2 showed statistically significant positive correlations between CSDxAPI, RSDxAPI, PNEUxAPI, and COPDxAPI, *r* > 0.50. The results indicate that the warmest, driest, and most polluted months of the fire season, represented by Cluster 2, are positively correlated with increase in cardio‐respiratory mortality rates. By considering a broader range of variables (mixture of air pollutants and meteorological variables) that could represent the atmospheric conditions at the surface level, this study revealed previously unrecognized associations and developed a nuanced understanding of the complex interactions between environmental factors and health outcomes.

Studies such as Baccini et al. (2008), S. Lin et al. (2009), H. Lin et al. (2013), Yang et al. (2012), and Vitolo et al. (2019) reported the association between combined hazards (elevated temperature—heat stress—and fire danger) and adverse health outcomes such as cardiovascular and respiratory diseases. Our work shows that the atmospheric conditions related to high temperatures, low relative humidity, and high near‐surface O_3_ concentration is associated with an increase in cardio‐respiratory mortality and contributes to an increase in the overall burden of disease. The API index has also been linked to dust aerosols. Dust aerosols play an important role in Europe due to dust storms from the Sahara Desert, many of them considered extreme events (Valenzuela et al., 2017). Studies show that cardiovascular hospitalizations increase after African dust storm episodes (Middleton et al., 2008; Neophytou et al., 2013).

The PBI and API indices were used to access monthly surface atmospheric conditions during the 2011–2020 wildfire seasons in Portugal and their potential impact on cardio‐respiratory mortality. According to Vitolo et al. (2019) because multiple hazards affecting the same region simultaneously can have significant impacts, the consequences of one hazard event are often exacerbated by interactions with another. This suggests the need for spatiotemporal information layers that identify hotspots of combined hazards (Vitolo et al., 2019). Here, we used multivariate statistical methods to create two fire‐pollutant meteorological indices (PBI and API) from different environmental variables to understand how the combination of these variables is related to cardio‐respiratory mortality rates. The results show that reducing the dimensionality of the database through PCs linear regression analysis efficiently helps to understand how fire‐pollutant meteorology indices can affect mortality rates.

By considering the combined effects of multiple pollutants‐atmospheric variables through fire‐pollutant‐meteorological factors and providing an accurate representation of health risks, the PBI and API indices offer a comprehensive and reliable assessment of the overall impact of adverse atmospheric conditions on public health in Portugal. The development and improvement of environmental health indices, such as the PBI and API, can greatly contribute to raising public awareness and facilitating informed decision‐making regarding adverse atmospheric conditions and its potential consequences for public health.

Importantly, however, this study also has some limitations. When examining the relationship between fire‐pollutant‐atmospheric variables and health outcomes, it is important to consider and account for potential confounding factors such as socioeconomic factors, population density, and individual health behaviors. Socioeconomic factors such as income, education level, access to health care, and living conditions can influence health outcomes, and population density in fire‐prone areas may influence exposure levels. Higher population density may lead to increased exposure to fire events and associated pollutants. On the other hand, individual health behaviors such as smoking habits, physical activity, and access to health services may potentially modify the associations between exposure and subsequent health outcomes. It is worth noting that identifying and appropriately accounting for all confounding factors can be challenging, and residual confounding may persist despite rigorous efforts. It is also important to recognize that correlation does not necessarily imply causation. There may be other factors at play that are not accounted for in the analysis. Another important information is that different health risks from fire‐pollutant‐meteorological factors may vary from area to area, and thus the construction of pollutant‐atmosphere interaction indices may need to account for these regional differences. Air pollution sources, population characteristics, climate, and other local factors may influence the specific health risks associated with air pollution in a given region, requiring model adjustments.

## 5 Conclusion

With climate change, extreme weather events and uncontrolled wildfires tend to become more frequent. Thus, morbidity and mortality tend to increase if mitigation measures are not taken. Portugal is a highly fire‐prone region and frequently suffers with intense natural hazards such as droughts, heat waves, and wildfires. Wildfires release great quantities of pollutants to the atmosphere which are a risk factor for adverse cardiovascular outcomes. A shared understanding of the health effects of fire, pollutants, and meteorology can help society and decision makers to be better prepared for extreme weather events and ensure that health services are able to mitigate public health consequences. Since studies of fire‐pollutant‐meteorology‐health relationships in Portugal are still scarce, this work suggests a novel methodology to combine fire‐pollutant‐meteorological variables and correlate them with health outcomes. For this study, it was used fire‐pollutant‐meteorology‐health data for the months of the fire season (June–July–August–September–October) of 2011–2020 in Portugal with a burned area greater than 1,000 ha.

In this study, PCA was used to reduce the dimensionality of data related to fire‐pollutant‐meteorological variables. The first two PCs (PC1 and PC2) were used to create two pollutant‐atmosphere interaction indices, PBI and API, which were highly correlated with air pollutants, meteorological variables, and burned area. The PBI and API indices were then used to cluster data points into two clusters based on their similarity in pollutant‐atmosphere interaction patterns. The study found statistically significant correlations between the indices and cardio‐respiratory mortality in Portugal during the wildfire seasons through a linear regression model. The results showed that months with extreme weather conditions and higher pollutant concentrations were positively correlated with an increase in cardio‐respiratory mortality rates. The study provides a nuanced understanding of the complex interactions between environmental factors and health outcomes.

It is recommended that future research expand the scope of the study by including additional variables such as population density, socioeconomic status, or other environmental factors that could potentially influence outcomes. It is also recommended that controlled experiments be designed to test specific hypotheses and establish causal relationships. Factors such as age, gender, or pre‐existing health conditions may influence the effects of wildfire, and studying these subgroups may provide valuable insights.

## Conflict of Interest

The authors declare no conflicts of interest relevant to this study.

## Supporting information

Supporting Information S1

## Data Availability

Most of the data for this study are publicly available online or must be requested from the appropriate agencies. Air pollution data of PM_10_, PM_2.5_, CO, O_3_, NO_2_ concentrations were obtained from the online air quality database (QualAr) of the Portuguese Environmental Agency (APA) at https://qualar.apambiente.pt/downloads; *Ano > Tipo de Estação*; choose the variables, the years, the months and the area shown at item Section 2.2. Characteristics of QualAr air quality stations used on this work are available at Table S2 in Supporting Information S1. Mortality data of CSD, RSD, PNEU, COPD, and ASMA for Portugal were provided by the National Institute of Statistics (INE; https://www.ine.pt/; for monthly mortality data, see *Products* > *Database* > *Theme:Health* > *Sub theme: Mortality by cause of death*; choose the variables, the years and the months shown at item Section 2.2). Meteorological data of temperature, relative humidity, and wind speed were provided by the Portuguese Institute of Sea and Atmosphere (IPMA; https://www.ipma.pt/pt/index.html). Characteristics of IPMA meteorological stations used on this work are available at Table S3 in Supporting Information S1. Burned area data were provided by the Portuguese Institute of Nature and Forest Conservation (ICNF; https://www.icnf.pt/). Burned area data must be requested at https://www.ipma.pt/pt/index.html since are not available for the public. Copernicus Atmosphere Monitoring Service (CAMS) data of AOD, BC_AOD and Dust_AOD are available through the Copernicus Atmosphere Monitoring Service (CAMS; https://ads.atmosphere.copernicus.eu/cdsapp#!/dataset/cams-global-reanalysis-eac4-monthly?tab=overview; choose the variables, the years, the months and the area shown at item Section 2.2). PBI‐API indices as well as cardio‐respiratory mortality normalized data used on this work are available at Table S4 in Supporting Information S1. The database (Duarte_et_al_Data set_GeoHealth.csv) used for multivariate statistical methods to investigate the effects of fire‐pollution‐meteorological variables on health outcomes in the study data are published on Zenodo Repository via https://doi.org/10.5281/zenodo.8086284 (E. Duarte, 2023).
