# Supplementary material for: Fire‐Pollutant‐Atmosphere Components and Its Impact on Mortality in Portugal During Wildfire Seasons
Source: Geohealth. 2023 Oct 6;7(10):e2023GH000802. doi: 10.1029/2023GH000802 (PMC10558046; doi:10.1029/2023GH000802)
Supplement: Supplementary file 1 — Supporting Information S1 [file GH2-7-e2023GH000802-s001.docx]

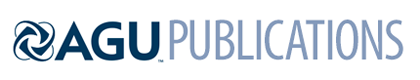


***GeoHealth***

Supporting Information for

**Fire-Pollutant-Atmosphere Components and Its Impact on Mortality in Portugal During Wildfire Seasons**

**Ediclê de Souza Fernandes Duarte^1,2,3,*^ , Vanda Salgueiro^1,2,3^, Maria João Costa^1,2,3^, Paulo Sérgio Lucio^4^, Miguel Potes^1,2,3^, Daniele Bortoli^1,2,3^, Rui Salgado^1,2,3^**

^1^Instituto de Ciências da Terra – ICT (Pólo de Évora), Instituto de Investigação e Formação Avançada (IIFA), Universidade de Évora, 7000-671 Évora, Portugal

^2^Earth Remote Sensing Laboratory (EaRSLab), Instituto de Investigação e Formação Avançada (IIFA), Universidade de Évora, Évora, Portugal

^3^Departamento de Física, Escola de Ciências e Tecnologia (ECT), Universidade de Évora, Évora, Portugal

^4^Departamento de Ciências Atmosféricas e Climáticas, Universidade Federal do Rio Grande do Norte, Natal, RN, Brazil

* Corresponding author: Ediclê Duarte [edicle.duarte@uevora.pt](mailto:edicle.duarte@uevora.pt)

**Appendix A. Additional Supporting Information S1**

**Table S1:** Data used on this work are publicly available online or must be requested from the appropriate agencies. Air pollution of PM_10_, PM_2.5_, CO, O_3_, NO_2_ concentrations were obtained from the online air quality database (QualAr) of the Portuguese Environmental Agency (APA) at <https://qualar.apambiente.pt/downloads>; chose the following options *Ano>Tipo de Estação*; choose the variables, the years, the months and the area shown at item **2.2. Burned area, air pollution and meteorological data**). Mortality data of CSD, RSD, PNEU, COPD and ASMA for Portugal were provided by the National Institute of Statistics (INE; <https://www.ine.pt/> ; for monthly mortality data, see *Products>Database>Theme:Health>Sub theme: Mortality by cause of death*; choose the variables, the years and the months shown at item **2.2. Burned area, air pollution and meteorological data**). Meteorological data of temperature, relative humidity and wind speed, were provided by the Portuguese Institute of Sea and Atmosphere (IPMA; <https://www.ipma.pt/pt/index.html>). Burned area data were provided by the Portuguese Institute of Nature and Forest Conservation (ICNF; <https://www.icnf.pt/>). Burned area data must be requested at <https://www.ipma.pt/pt/index.html> since are not available for the public. Copernicus Atmosphere Monitoring Service (CAMS) data of AOD, BC_AOD and Dust_AOD are available through the Copernicus Atmosphere Monitoring Service (CAMS; <https://ads.atmosphere.copernicus.eu/cdsapp#!/dataset/cams-global-reanalysis-eac4-monthly?tab=overview> ; choose the variables, the years, the months and the area shown at item **2.2. Burned area, air pollution and meteorological data**).

| **Date** | **Cluster** | | **PM10_Obs** | | | **PM25_Obs** | | **CO_Obs** | | **O3_Obs** | | | **NO2_Obs** | | | **WHO_PM10** | | | | **WHO_PM25** | |
| --- | --- | --- | --- | --- | --- | --- | --- | --- | --- | --- | --- | --- | --- | --- | --- | --- | --- | --- | --- | --- | --- |
| Jun-11 | **2** | | 23.58 | | | 7.01 | | 0.21 | | 62.41 | | | 8.88 | | | 2 | | | | 2 | |
| Jul-11 | **2** | | 22.46 | | | 6.74 | | 0.18 | | 55.73 | | | 8.09 | | | 0 | | | | 0 | |
| Aug-11 | **2** | | 20.71 | | | 6.62 | | 0.18 | | 49.3 | | | 10.58 | | | 0 | | | | 1 | |
| Sep-11 | **1** | | 24.17 | | | 7.26 | | 0.23 | | 49.23 | | | 16.4 | | | 1 | | | | 1 | |
| Oct-11 | **1** | | 40.79 | | | 14.32 | | 0.36 | | 57.91 | | | 19.07 | | | 11 | | | | 14 | |
| Jun-12 | **2** | | 18.71 | | | 5.92 | | 0.14 | | 55.28 | | | 9.32 | | | 1 | | | | 2 | |
| Jul-12 | **2** | | 20.45 | | | 6.82 | | 0.19 | | 60.33 | | | 9.7 | | | 0 | | | | 2 | |
| Aug-12 | **1** | | 17.84 | | | 5.26 | | 0.22 | | 49.84 | | | 11.04 | | | 0 | | | | 0 | |
| Sep-12 | **1** | | 25.37 | | | 8.26 | | 0.26 | | 59.05 | | | 14.46 | | | 0 | | | | 0 | |
| Jun-13 | **2** | | 17.91 | | | 8.55 | | 0.32 | | 70.59 | | | 8.79 | | | 0 | | | | 1 | |
| Jul-13 | **2** | | 22.02 | | | 10.77 | | 0.38 | | 76.82 | | | 10.23 | | | 0 | | | | 6 | |
| Aug-13 | **2** | | 25.43 | | | 10.54 | | 0.46 | | 67.18 | | | 11.03 | | | 0 | | | | 5 | |
| Sep-13 | **1** | | 25.49 | | | 10.93 | | 0.48 | | 60.66 | | | 14.15 | | | 1 | | | | 8 | |
| Jun-14 | **1** | | 17.32 | | | 6.7 | | 0.17 | | 64.97 | | | 8.99 | | | 0 | | | | 0 | |
| Jul-14 | **2** | | 18.16 | | | 7.26 | | 0.17 | | 61.76 | | | 9.05 | | | 0 | | | | 0 | |
| Aug-14 | **1** | | 14.75 | | | 5.29 | | 0.17 | | 52.14 | | | 8.46 | | | 0 | | | | 0 | |
| Sep-14 | **1** | | 16.78 | | | 5.97 | | 0.22 | | 47.16 | | | 12.75 | | | 0 | | | | 0 | |
| Jun-15 | **2** | | 20.95 | | | 10.23 | | 0.18 | | 69.19 | | | 12.23 | | | 0 | | | | 4 | |
| Jul-15 | **2** | | 16.41 | | | 7.76 | | 0.17 | | 49.04 | | | 8.87 | | | 0 | | | | 0 | |
| Aug-15 | **2** | | 17.03 | | | 8.21 | | 0.19 | | 50.58 | | | 10.46 | | | 0 | | | | 3 | |
| Sep-15 | **1** | | 18.86 | | | 8.73 | | 0.2 | | 57.85 | | | 14.86 | | | 0 | | | | 1 | |
| Oct-15 | **1** | | 18.70 | | | 8.34 | | 0.22 | | 48.94 | | | 15.79 | | | 0 | | | | 3 | |
| Jul-16 | **2** | | 20.45 | | | 8.11 | | 0.17 | | 62.56 | | | 11.58 | | | 0 | | | | 0 | |
| Aug-16 | **2** | | 25.91 | | | 10.13 | | 0.2 | | 63.97 | | | 11.73 | | | 4 | | | | 5 | |
| Sep-16 | **1** | | 19.54 | | | 7.35 | | 0.18 | | 52.87 | | | 13.29 | | | 1 | | | | 2 | |
| Oct-16 | **1** | | 20.85 | | | 7.7 | | 0.21 | | 48.78 | | | 15.87 | | | 2 | | | | 2 | |
| Jun-17 | **2** | | 21.56 | | | 9.23 | | 0.19 | | 58.5 | | | 8.77 | | | 0 | | | | 4 | |
| Jul-17 | **2** | | 16.00 | | | 7.9 | | 0.18 | | 54.41 | | | 8.94 | | | 0 | | | | 0 | |
| Aug-17 | **2** | | 18.08 | | | 9.85 | | 0.2 | | 53.85 | | | 10.91 | | | 0 | | | | 4 | |
| Sep-17 | **1** | | 16.48 | | | 7.3 | | 0.21 | | 51.13 | | | 12.01 | | | 0 | | | | 0 | |
| Oct-17 | **1** | | 29.31 | | | 11.95 | | 0.24 | | 49.97 | | | 21.67 | | | 2 | | | | 9 | |
| Aug-18 | **2** | | 24.02 | | | 11.38 | | 0.24 | | 65.12 | | | 13.4 | | | 2 | | | | 5 | |
| Sep-18 | **1** | | 22.14 | | | 11.42 | | 0.27 | | 63.33 | | | 16.32 | | | 0 | | | | 3 | |
| Oct-18 | **1** | | 19.25 | | | 8.15 | | 0.22 | | 53.98 | | | 18.47 | | | 0 | | | | 2 | |
| Jun-19 | **1** | | 13.09 | | | 4.89 | | 0.15 | | 60.33 | | | 9.88 | | | 0 | | | | 0 | |
| Jul-19 | **2** | | 19.04 | | | 7.95 | | 0.16 | | 57.93 | | | 10.12 | | | 0 | | | | 0 | |
| Aug-19 | **1** | | 15.59 | | | 6.07 | | 0.13 | | 54.78 | | | 9.81 | | | 0 | | | | 0 | |
| Sep-19 | **1** | | 20.08 | | | 7.36 | | 0.17 | | 57 | | | 14.8 | | | 0 | | | | 0 | |
| Jun-20 | **1** | | 12.02 | | | 5.13 | | 0.12 | | 56.96 | | | 6.52 | | | 0 | | | | 0 | |
| Jul-20 | **2** | | 18.97 | | | 8.56 | | 0.17 | | 64.69 | | | 10.5 | | | 0 | | | | 0 | |
| Aug-20 | **1** | | 12.58 | | | 4.79 | | 0.13 | | 51.03 | | | 6.97 | | | 0 | | | | 0 | |
| Sep-20 | **1** | | 17.13 | | | 6.66 | | 0.18 | | 61.83 | | | 12.69 | | | 0 | | | | 0 | |
| Oct-20 | **1** | | 14.06 | | | 5.09 | | 0.21 | | 50.76 | | | 11.91 | | | 0 | | | | 0 | |
|  | | **WHO_NO2** | | **TEMP_Obs** | | | **RH_Obs** | | **WS_Obs** | | | **Burned_Area** | | | | | **AOD_CAMs** | | | |  |
|  | | 0 | | 19.98 | | | 61.26 | | 2.91 | | | 3431.03 | | | | | 1.36E-01 | | | |  |
|  | | 0 | | 21.12 | | | 60.50 | | 3.33 | | | 12526.08 | | | | | 1.09E-01 | | | |  |
|  | | 0 | | 21.80 | | | 63.02 | | 2.72 | | | 19187.40 | | | | | 1.46E-01 | | | |  |
|  | | 4 | | 20.29 | | | 64.36 | | 2.26 | | | 6516.85 | | | | | 1.12E-01 | | | |  |
|  | | 6 | | 18.06 | | | 60.01 | | 2.58 | | | 28972.74 | | | | | 1.49E-01 | | | |  |
|  | | 1 | | 19.66 | | | 65.36 | | 3.14 | | | 1208.38 | | | | | 1.91E-01 | | | |  |
|  | | 0 | | 21.32 | | | 58.57 | | 3.08 | | | 35890.29 | | | | | 1.13E-01 | | | |  |
|  | | 0 | | 21.70 | | | 61.03 | | 2.84 | | | 7522.18 | | | | | 9.90E-02 | | | |  |
|  | | 1 | | 20.74 | | | 60.95 | | 2.65 | | | 37391.58 | | | | | 1.45E-01 | | | |  |
|  | | 0 | | 15.48 | | | 77.65 | | 2.31 | | | 721.33 | | | | | 1.26E-01 | | | |  |
|  | | 0 | | 19.25 | | | 59.53 | | 3.04 | | | 2751.10 | | | | | 1.96E-01 | | | |  |
|  | | 0 | | 23.25 | | | 58.62 | | 2.48 | | | 22319.59 | | | | | 1.62E-01 | | | |  |
|  | | 0 | | 23.31 | | | 54.58 | | 2.67 | | | 102698.54 | | | | | 1.37E-01 | | | |  |
|  | | 0 | | 21.19 | | | 61.41 | | 2.54 | | | 26316.77 | | | | | 1.15E-01 | | | |  |
|  | | 0 | | 16.51 | | | 79.02 | | 2.39 | | | 745.74 | | | | | 1.10E-01 | | | |  |
|  | | 0 | | 19.13 | | | 65.29 | | 2.85 | | | 2452.24 | | | | | 8.87E-02 | | | |  |
|  | | 0 | | 21.25 | | | 64.72 | | 2.90 | | | 2532.86 | | | | | 1.20E-01 | | | |  |
|  | | 0 | | 21.18 | | | 63.90 | | 2.96 | | | 8569.88 | | | | | 1.57E-01 | | | |  |
|  | | 0 | | 19.69 | | | 77.34 | | 2.34 | | | 4891.28 | | | | | 1.04E-01 | | | |  |
|  | | 0 | | 18.09 | | | 76.20 | | 2.45 | | | 430.16 | | | | | 1.22E-01 | | | |  |
|  | | 1 | | 21.61 | | | 58.02 | | 2.64 | | | 5185.24 | | | | | 1.03E-01 | | | |  |
|  | | 3 | | 23.05 | | | 59.90 | | 3.03 | | | 11124.84 | | | | | 1.03E-01 | | | |  |
|  | | 0 | | 21.76 | | | 61.28 | | 2.82 | | | 29912.90 | | | | | 1.11E-01 | | | |  |
|  | | 0 | | 18.71 | | | 65.00 | | 2.53 | | | 4343.47 | | | | | 1.92E-01 | | | |  |
|  | | 0 | | 16.15 | | | 79.03 | | 2.81 | | | 1528.24 | | | | | 1.11E-01 | | | |  |
|  | | 1 | | 20.18 | | | 64.08 | | 3.00 | | | 675.12 | | | | | 1.22E-01 | | | |  |
|  | | 0 | | 24.06 | | | 54.03 | | 2.85 | | | 7825.38 | | | | | 1.98E-01 | | | |  |
|  | | 0 | | 23.88 | | | 52.66 | | 2.77 | | | 118836.43 | | | | | 1.18E-01 | | | |  |
|  | | 0 | | 20.89 | | | 60.48 | | 2.56 | | | 34331.71 | | | | | 1.35E-01 | | | |  |
|  | | 0 | | 16.72 | | | 74.12 | | 2.29 | | | 4007.21 | | | | | 1.07E-01 | | | |  |
|  | | 10 | | 22.13 | | | 59.44 | | 3.02 | | | 56837.83 | | | | | 2.08E-01 | | | |  |
|  | | 1 | | 22.60 | | | 57.72 | | 3.07 | | | 74503.38 | | | | | 2.01E-01 | | | |  |
|  | | 1 | | 22.79 | | | 55.01 | | 2.86 | | | 89976.73 | | | | | 1.52E-01 | | | |  |
|  | | 5 | | 19.52 | | | 60.12 | | 2.72 | | | 13367.30 | | | | | 9.90E-02 | | | |  |
|  | | 0 | | 19.04 | | | 57.12 | | 2.17 | | | 289126.35 | | | | | 9.18E-02 | | | |  |
|  | | 0 | | 18.97 | | | 72.30 | | 2.74 | | | 236.24 | | | | | 1.60E-01 | | | |  |
|  | | 0 | | 20.52 | | | 70.91 | | 2.78 | | | 593.99 | | | | | 1.10E-01 | | | |  |
|  | | 0 | | 24.23 | | | 53.56 | | 2.67 | | | 31162.84 | | | | | 1.22E-01 | | | |  |
|  | | 0 | | 22.53 | | | 60.21 | | 2.25 | | | 3716.25 | | | | | 1.22E-01 | | | |  |
|  | | 0 | | 16.03 | | | 67.44 | | 2.81 | | | 3890.28 | | | | | 9.54E-02 | | | |  |
|  | | 0 | | 17.97 | | | 64.84 | | 3.07 | | | 3001.03 | | | | | 7.83E-02 | | | |  |
|  | | 0 | | 21.71 | | | 64.87 | | 2.97 | | | 14111.36 | | | | | 1.40E-01 | | | |  |
|  | | 0 | | 22.04 | | | 62.41 | | 2.89 | | | 4612.73 | | | | | 1.01E-01 | | | |  |
|  | | **BC_AOD_CAMs** | | | **Dust_AOD_CAMs** | | | | **CSD** | | **RSD** | | | **PNEU** | **COPD** | | | **ASMA** |  |  |  |
|  | | 9.99E-03 | | | 1.78E-02 | | | | 21.892 | | 7.119 | | | 3.176 | 1.554 | | | 0.078 |  |  |  |
|  | | 8.32E-03 | | | 1.31E-02 | | | | 21.834 | | 7.430 | | | 3.370 | 1.554 | | | 0.058 |  |  |  |
|  | | 5.65E-03 | | | 4.99E-02 | | | | 22.261 | | 8.023 | | | 3.992 | 1.437 | | | 0.039 |  |  |  |
|  | | 7.13E-03 | | | 5.88E-03 | | | | 21.785 | | 7.042 | | | 3.273 | 1.350 | | | 0.068 |  |  |  |
|  | | 1.09E-02 | | | 5.35E-03 | | | | 23.135 | | 8.295 | | | 3.992 | 1.826 | | | 0.068 |  |  |  |
|  | | 8.51E-03 | | | 5.10E-02 | | | | 21.445 | | 8.372 | | | 4.031 | 1.981 | | | 0.087 |  |  |  |
|  | | 7.70E-03 | | | 1.16E-02 | | | | 22.261 | | 8.052 | | | 3.866 | 1.544 | | | 0.117 |  |  |  |
|  | | 5.52E-03 | | | 2.11E-02 | | | | 22.242 | | 7.498 | | | 3.914 | 1.311 | | | 0.039 |  |  |  |
|  | | 1.04E-02 | | | 1.02E-02 | | | | 19.445 | | 7.848 | | | 4.002 | 1.593 | | | 0.078 |  |  |  |
|  | | 9.52E-03 | | | 3.99E-03 | | | | 22.514 | | 8.499 | | | 4.128 | 1.544 | | | 0.039 |  |  |  |
|  | | 1.79E-02 | | | 1.66E-02 | | | | 24.855 | | 10.975 | | | 5.255 | 2.166 | | | 0.117 |  |  |  |
|  | | 1.73E-02 | | | 9.84E-03 | | | | 22.368 | | 8.285 | | | 4.011 | 1.729 | | | 0.097 |  |  |  |
|  | | 1.05E-02 | | | 3.79E-03 | | | | 20.474 | | 8.333 | | | 4.099 | 1.632 | | | 0.068 |  |  |  |
|  | | 6.37E-03 | | | 7.17E-03 | | | | 23.009 | | 7.751 | | | 3.759 | 1.593 | | | 0.049 |  |  |  |
|  | | 7.52E-03 | | | 8.81E-03 | | | | 22.067 | | 7.848 | | | 3.351 | 1.768 | | | 0.049 |  |  |  |
|  | | 7.40E-03 | | | 7.69E-03 | | | | 22.193 | | 7.809 | | | 3.730 | 1.554 | | | 0.078 |  |  |  |
|  | | 4.93E-03 | | | 5.61E-03 | | | | 22.135 | | 7.440 | | | 3.390 | 1.573 | | | 0.097 |  |  |  |
|  | | 8.27E-03 | | | 2.03E-02 | | | | 21.882 | | 7.867 | | | 3.642 | 1.700 | | | 0.039 |  |  |  |
|  | | 8.43E-03 | | | 1.49E-02 | | | | 21.183 | | 7.556 | | | 3.186 | 1.573 | | | 0.087 |  |  |  |
|  | | 6.55E-03 | | | 1.91E-02 | | | | 21.212 | | 6.925 | | | 2.962 | 1.418 | | | 0.039 |  |  |  |
|  | | 6.55E-03 | | | 1.74E-03 | | | | 21.931 | | 7.207 | | | 3.351 | 1.428 | | | 0.049 |  |  |  |
|  | | 2.57E-03 | | | 7.72E-03 | | | | 22.727 | | 8.246 | | | 3.613 | 1.894 | | | 0.087 |  |  |  |
|  | | 5.26E-03 | | | 2.22E-02 | | | | 23.417 | | 9.402 | | | 4.312 | 1.816 | | | 0.107 |  |  |  |
|  | | 1.69E-02 | | | 2.03E-02 | | | | 23.029 | | 8.567 | | | 3.778 | 1.758 | | | 0.049 |  |  |  |
|  | | 6.10E-03 | | | 1.69E-02 | | | | 21.747 | | 7.809 | | | 3.127 | 1.816 | | | 0.136 |  |  |  |
|  | | 4.44E-03 | | | 1.02E-02 | | | | 23.611 | | 8.547 | | | 3.584 | 1.884 | | | 0.117 |  |  |  |
|  | | 1.12E-02 | | | 3.77E-02 | | | | 23.213 | | 8.460 | | | 3.662 | 1.923 | | | 0.126 |  |  |  |
|  | | 5.88E-03 | | | 2.61E-02 | | | | 21.620 | | 7.401 | | | 3.157 | 1.447 | | | 0.029 |  |  |  |
|  | | 1.10E-02 | | | 1.86E-02 | | | | 21.785 | | 7.566 | | | 3.205 | 1.535 | | | 0.039 |  |  |  |
|  | | 1.04E-02 | | | 7.27E-03 | | | | 20.911 | | 7.139 | | | 3.059 | 1.457 | | | 0.058 |  |  |  |
|  | | 2.78E-02 | | | 2.95E-02 | | | | 23.495 | | 8.877 | | | 4.070 | 1.651 | | | 0.087 |  |  |  |
|  | | 1.20E-02 | | | 5.85E-02 | | | | 24.709 | | 8.722 | | | 3.827 | 1.807 | | | 0.068 |  |  |  |
|  | | 9.88E-03 | | | 1.29E-02 | | | | 20.843 | | 7.178 | | | 3.089 | 1.690 | | | 0.078 |  |  |  |
|  | | 3.23E-03 | | | 1.06E-02 | | | | 22.747 | | 8.120 | | | 3.467 | 1.797 | | | 0.097 |  |  |  |
|  | | 5.85E-03 | | | 6.02E-03 | | | | 22.912 | | 7.556 | | | 2.710 | 1.797 | | | 0.087 |  |  |  |
|  | | 7.79E-03 | | | 2.79E-02 | | | | 23.330 | | 7.265 | | | 2.438 | 1.768 | | | 0.117 |  |  |  |
|  | | 6.34E-03 | | | 4.85E-03 | | | | 23.165 | | 6.945 | | | 2.613 | 1.505 | | | 0.078 |  |  |  |
|  | | 5.25E-03 | | | 2.75E-02 | | | | 22.446 | | 6.614 | | | 2.584 | 1.418 | | | 0.058 |  |  |  |
|  | | 5.00E-03 | | | 8.68E-03 | | | | 24.136 | | 6.216 | | | 2.292 | 1.690 | | | 0.165 |  |  |  |
|  | | 3.36E-03 | | | 3.07E-02 | | | | 27.749 | | 9.382 | | | 3.652 | 1.807 | | | 0.126 |  |  |  |
|  | | 3.49E-03 | | | 9.51E-03 | | | | 24.660 | | 7.071 | | | 2.516 | 2.011 | | | 0.146 |  |  |  |
|  | | 9.22E-03 | | | 9.82E-03 | | | | 24.233 | | 7.605 | | | 2.836 | 1.816 | | | 0.146 |  |  |  |
|  | | 7.46E-03 | | | 1.67E-03 | | | | 26.224 | | 7.508 | | | 2.846 | 1.603 | | | 0.068 |  |  |  |

**Table S2**: Characteristics of QualAr air quality stations used on this work. Data can be downloaded from the Portuguese Environmental Agency (APA) at <https://qualar.apambiente.pt/downloads>.

| Tipo | Area | Station | LATITUDE | LONGITUDE |
| --- | --- | --- | --- | --- |
| Background | Urban | Alfragide_Amadora | 38.7548 | -9.23002 |
| Background | Suburban | Anta_Espinho | 40.9995 | -8.62328 |
| Background | Urban | Arcos | 38.5296 | -8.89313 |
| Background | Urban | Avintes | 41.0982 | -8.55435 |
| Background | Urban | Beato | 38.7337 | -9.1145 |
| Background | Urban | Burgães | 41.3538 | -8.46058 |
| Background | Urban | Cascais | 38.7014 | -9.42925 |
| Background | Rural | Cerro | 37.3125 | -7.67861 |
| Background | Rural | Chamusca | 39.3541 | -8.4674 |
| Background | Suburban | Custóias-Matosinhos | 41.1998 | -8.64484 |
| Background | Rural | Douro_Norte | 41.3713 | -7.79082 |
| Background | Urban | Ermesinde | 41.2066 | -8.5526 |
| Background | Rural | Ervedeira | 39.9246 | -8.89294 |
| Background | Urban | Escavadeira | 38.6609 | -9.068 |
| Background | Suburban | Estarreja | 40.7586 | -8.56716 |
| Background | Rural | Fernando_Po | 38.6373 | -8.69176 |
| Background | Urban | Fidalguinho | 38.6501 | -9.04868 |
| Background | Rural | Fornelo_Monte | 40.6441 | -8.10001 |
| Background | Suburban | Frossos_Braga | 41.5695 | -8.45696 |
| Background | Rural | Fundao | 40.2331 | -7.29959 |
| Background | Suburban | Ilhavo | 40.5909 | -8.67203 |
| Background | Urban | ICG | 40.2085 | -8.41217 |
| Background | Urban | Joaquim_Magalhaes | 37.015 | -7.92667 |
| Background | Urban | Laranjeiro | 38.6635 | -9.15764 |
| Background | Suburban | Leca_do_Balio | 41.2181 | -8.63217 |
| Background | Suburban | Lourinha | 39.28 | -9.24704 |
| Background | Urban | Malpique | 37.0917 | -8.24972 |
| Background | Urban | Mem_Martins | 38.7865 | -9.34854 |
| Background | Suburban | Mindelo_Vila | 41.3084 | -8.72085 |
| Background | Rural | Minho_Lima | 41.8021 | -8.69385 |
| Background | Rural | Monte_Velho | 38.077 | -8.7986 |
| Background | Rural | Montemor | 40.2022 | -8.66889 |
| Background | Urban | Olivais | 38.7698 | -9.10729 |
| Background | Urban | Pacos_de_Ferreira | 41.2741 | -8.37586 |
| Background | Urban | Quinta_do_Marques | 38.6983 | -9.32248 |
| Background | Urban | Reboleira | 38.7548 | -9.23002 |
| Background | Urban | Restelo | 38.7057 | -9.20946 |
| Background | Rural | Santa_Combinha | 41.5719 | -6.88722 |
| Background | Urban | Sobreiras_Lordelo | 41.1474 | -8.65897 |
| Background | Rural | Terena | 38.6168 | -7.39889 |
| Background | Suburban | VNTelha_Maia | 41.2523 | -8.66049 |

**Table S3**: Characteristics of IPMA meteorological stations used on this work. Data for these stations must be requested at <https://www.ipma.pt/pt/siteinfo/contacto.jsp> since are not available for the public assess.

| Num_Est | NomeEstacao | LATITUDE | LONGITUDE | Altitude |
| --- | --- | --- | --- | --- |
| 1200535 | Lisboa / Geofísico | 38.719 | -9.150 | 77.000 |
| 1200536 | Lisboa - Portela | 38.789 | -9.135 | 103.000 |
| 1200545 | Porto / Pedras Rubras | 41.232 | -8.679 | 67.726 |
| 1200548 | Coimbra / Aeródromo | 40.158 | -8.469 | 170.786 |
| 1200554 | Faro / Aeroporto | 37.017 | -7.972 | 5.003 |
| 1200558 | Évora / Aeródromo | 38.537 | -7.888 | 247.558 |
| 1200559 | Viseu/CC | 40.726 | -7.887 | 628.000 |
| 1200562 | Beja | 38.026 | -7.867 | 246.000 |
| 1200567 | Vila Real/Aeródromo | 41.274 | -7.717 | 561.475 |
| 1200568 | Penhas Douradas / Observatório | 40.411 | -7.559 | 1,380.000 |
| 1200571 | Portalegre | 39.294 | -7.421 | 597.000 |
| 1200576 | Bragança | 41.857 | -6.706 | 687.000 |
| 1200579 | Lisboa/Gago Coutinho | 38.766 | -9.128 | 103.884 |
| 1210577 | Odemira/S.Teotónio | 37.547 | -8.729 | 120.544 |
| 1210580 | Lisboa Gago Coutinho | 38.762 | -9.125 | 114.098 |
| 1210604 | Vila Nova de Cerveira / Aeródromo | 41.973 | -8.676 | 34.000 |
| 1210611 | Montalegre | 41.823 | -7.788 | 1,005.000 |
| 1210612 | Vinhais | 41.843 | -7.003 | 773.000 |
| 1210616 | Chaves / Aeródromo | 41.725 | -7.465 | 353.350 |
| 1210625 | Guimarães | 41.429 | -8.318 | 173.967 |
| 1210632 | Mirandela | 41.515 | -7.191 | 250.000 |
| 1210655 | Pinhão | 41.173 | -7.549 | 130.000 |
| 1210665 | Mêda | 40.969 | -7.257 | 700.000 |
| 1210666 | Trancoso / Bandarra | 40.781 | -7.357 | 840.000 |
| 1210683 | Guarda | 40.529 | -7.279 | 1,001.000 |
| 1210685 | Nelas | 40.523 | -7.855 | 425.000 |
| 1210686 | Pampilhosa da Serra | 40.145 | -7.927 | 835.588 |
| 1210687 | Covilhã | 40.264 | -7.482 | 482.000 |
| 1210698 | Fundão | 40.141 | -7.504 | 493.000 |
| 1210702 | Aveiro / Universidade | 40.635 | -8.660 | 3.631 |
| 1210707 | Coimbra / Bencanta | 40.213 | -8.455 | 26.618 |
| 1210718 | Leiria / Aeródromo | 39.781 | -8.821 | 42.523 |
| 1210734 | Santarém / Fonte Boa Est. Zootécnica | 39.201 | -8.737 | 71.911 |
| 1210735 | Chamusca / Chouto | 39.270 | -8.351 | 115.000 |
| 1210767 | Pegões | 38.651 | -8.635 | 64.000 |
| 1210837 | Estremoz / Techocas | 38.862 | -7.513 | 366.000 |
| 1210847 | Viana do Alentejo | 38.339 | -8.047 | 201.828 |
| 1210840 | Reguengos / S.Pedro do Corval | 38.485 | -7.473 | 265.168 |
| 1210848 | Portel / Oriola | 38.318 | -7.861 | 200.019 |
| 1210851 | Amareleja | 38.201 | -7.226 | 182.977 |
| 1210863 | Mértola / Vale Formoso | 37.758 | -7.552 | 199.507 |
| 1210866 | Vila Real de Santo António | 37.187 | -7.417 | 4.770 |
| 1210878 | Portimão / Aeródromo | 37.147 | -8.583 | 2.000 |

**Table S4**: PBI-API indices as well as cardio-respiratory mortality normalized data used on this in PCRL.

| **Date** | **Cluster** | **PBI** | **API** | **CSD** | **RSD** | **PNEU** | **COPD** | **ASMA** |
| --- | --- | --- | --- | --- | --- | --- | --- | --- |
| **Jun-11** | **2** | 1.499 | 4.360 | 0.364 | 0.567 | 1.615 | 0.422 | -1.886 |
| **Jul-11** | **2** | 0.649 | 4.830 | 0.364 | 0.569 | 1.729 | 0.422 | -2.037 |
| **Aug-11** | **2** | 1.185 | 4.531 | 0.364 | 0.571 | 2.075 | 0.350 | -2.222 |
| **Sep-11** | **1** | 1.820 | 0.355 | 0.364 | 0.567 | 1.672 | 0.291 | -1.957 |
| **Oct-11** | **1** | 3.750 | -0.371 | 0.364 | 0.572 | 2.075 | 0.567 | -1.957 |
| **Jun-12** | **2** | 1.199 | 4.987 | 0.364 | 0.572 | 2.096 | 0.639 | -1.828 |
| **Jul-12** | **2** | 1.090 | 4.807 | 0.364 | 0.571 | 2.007 | 0.416 | -1.661 |
| **Aug-12** | **1** | 0.528 | 3.540 | 0.364 | 0.569 | 2.033 | 0.264 | -2.222 |
| **Sep-12** | **1** | 1.902 | 3.241 | 0.364 | 0.571 | 2.080 | 0.445 | -1.886 |
| **Jun-13** | **2** | 1.343 | 4.842 | 0.364 | 0.573 | 2.147 | 0.416 | -2.222 |
| **Jul-13** | **2** | 2.682 | 6.892 | 0.364 | 0.578 | 2.710 | 0.716 | -1.661 |
| **Aug-13** | **2** | 2.757 | 6.311 | 0.364 | 0.572 | 2.085 | 0.519 | -1.768 |
| **Sep-13** | **1** | 2.612 | 2.987 | 0.364 | 0.572 | 2.132 | 0.467 | -1.957 |
| **Jun-14** | **1** | 0.550 | 3.514 | 0.364 | 0.570 | 1.948 | 0.445 | -2.118 |
| **Jul-14** | **2** | 0.672 | 4.074 | 0.364 | 0.571 | 1.718 | 0.539 | -2.118 |
| **Aug-14** | **1** | -0.151 | 3.447 | 0.364 | 0.570 | 1.932 | 0.422 | -1.886 |
| **Sep-14** | **1** | 0.564 | 0.413 | 0.364 | 0.569 | 1.740 | 0.433 | -1.768 |
| **Jun-15** | **2** | 1.931 | 5.287 | 0.364 | 0.571 | 1.883 | 0.503 | -2.222 |
| **Jul-15** | **2** | 0.606 | 4.645 | 0.364 | 0.569 | 1.621 | 0.433 | -1.828 |
| **Aug-15** | **2** | 1.180 | 3.905 | 0.364 | 0.566 | 1.486 | 0.337 | -2.222 |
| **Sep-15** | **1** | 1.306 | 1.412 | 0.364 | 0.568 | 1.718 | 0.344 | -2.118 |
| **Oct-15** | **1** | 1.016 | -0.781 | 0.364 | 0.572 | 1.867 | 0.600 | -1.828 |
| **Jul-16** | **2** | 1.201 | 5.901 | 0.364 | 0.575 | 2.244 | 0.562 | -1.712 |
| **Aug-16** | **2** | 2.769 | 6.741 | 0.364 | 0.573 | 1.959 | 0.534 | -2.118 |
| **Sep-16** | **1** | 1.369 | 2.862 | 0.364 | 0.570 | 1.586 | 0.562 | -1.571 |
| **Oct-16** | **1** | 1.441 | -0.515 | 0.364 | 0.573 | 1.851 | 0.595 | -1.661 |
| **Jun-17** | **2** | 1.952 | 6.679 | 0.364 | 0.573 | 1.894 | 0.613 | -1.617 |
| **Jul-17** | **2** | 0.925 | 5.602 | 0.364 | 0.569 | 1.603 | 0.356 | -2.349 |
| **Aug-17** | **2** | 1.860 | 5.424 | 0.364 | 0.569 | 1.632 | 0.411 | -2.222 |
| **Sep-17** | **1** | 1.002 | 2.792 | 0.364 | 0.567 | 1.545 | 0.363 | -2.037 |
| **Oct-17** | **1** | 3.904 | 1.073 | 0.364 | 0.574 | 2.117 | 0.477 | -1.828 |
| **Aug-18** | **2** | 2.703 | 7.425 | 0.364 | 0.573 | 1.986 | 0.558 | -1.957 |
| **Sep-18** | **1** | 2.321 | 3.462 | 0.364 | 0.568 | 1.563 | 0.498 | -1.886 |
| **Oct-18** | **1** | 1.433 | -0.365 | 0.364 | 0.572 | 1.784 | 0.553 | -1.768 |
| **Jun-19** | **1** | -0.557 | 2.850 | 0.364 | 0.569 | 1.328 | 0.553 | -1.828 |
| **Jul-19** | **2** | 1.116 | 4.927 | 0.364 | 0.568 | 1.150 | 0.539 | -1.661 |
| **Aug-19** | **1** | 0.239 | 3.800 | 0.364 | 0.566 | 1.265 | 0.393 | -1.886 |
| **Sep-19** | **1** | 1.166 | 3.265 | 0.364 | 0.565 | 1.247 | 0.337 | -2.037 |
| **Jun-20** | **1** | -0.927 | 3.275 | 0.364 | 0.562 | 1.051 | 0.498 | -1.451 |
| **Jul-20** | **2** | 1.133 | 6.340 | 0.364 | 0.575 | 1.889 | 0.558 | -1.617 |
| **Aug-20** | **1** | -1.581 | 3.782 | 0.364 | 0.567 | 1.202 | 0.652 | -1.527 |
| **Sep-20** | **1** | 1.247 | 3.541 | 0.364 | 0.570 | 1.408 | 0.562 | -1.527 |
| **Oct-20** | **1** | -0.151 | 0.051 | 0.364 | 0.569 | 1.414 | 0.450 | -1.957 |
